# Artificial intelligence for triple-negative breast cancer from imaging to multi-omics

**DOI:** 10.3389/fonc.2026.1834128

**Published:** 2026-06-30

**Authors:** Xing Peng, Xinyu Zhou, Xin Feng, Nimin Fang, Xiaoya Dong, Wanjing Hong, Tianli Li, Renxing Li, Mohammad Faidzul Nasrudin

**Affiliations:** 1 Center for Artificial Intelligence Technology, Universiti Kebangsaan Malaysia, Bangi, Malaysia; 2 School of Computer Science and Artificial Intelligence, Chaohu University, Hefei, China; 3 Department of Biomedical Engineering, Southern University of Science and Technology, Shenzhen, China

**Keywords:** artificial intelligence, breast ultrasound, digital pathology, magnetic resonance imaging, mammography, multimodal learning, multi-omics, triple-negative breast cancer

## Abstract

**Background:**

Triple-negative breast cancer (TNBC) is an aggressive and biologically heterogeneous breast cancer subtype for which robust biomarkers for diagnosis, treatment-response assessment, and prognosis remain limited. Artificial intelligence (AI) is increasingly used to analyze radiology, digital pathology, and molecular data in TNBC.

**Methods:**

This Review provides a structured narrative synthesis of previously published studies on AI for TNBC, with emphasis on imaging, computational pathology, genomics, multi-omics, and multimodal fusion. The literature was organized by data modality, clinical task, validation strategy, and translational readiness, with particular attention to external validation, calibration, interpretability, and missing-data handling.

**Results:**

Across modalities, AI has been applied to lesion segmentation, subtype classification, prediction of pathological complete response after neoadjuvant therapy, recurrence-risk stratification, and survival modeling. Magnetic resonance imaging, ultrasound, mammography, whole-slide histopathology, transcriptomics, and multi-omics provide complementary information, while multimodal fusion and radiogenomic frameworks appear most promising for capturing TNBC heterogeneity. However, the current evidence base is still limited by small cohorts, inconsistent endpoint definitions, non-patient-level splitting, inadequate external testing, and domain shift across scanners, stains, assays, and institutions.

**Discussion:**

The most clinically credible TNBC AI studies are those aligned with actionable clinical decisions and supported by robust validation, transparent reporting, and biologically grounded interpretation. Future progress will depend on multi-institutional data curation, self-supervised and foundation-model pretraining, privacy-preserving collaboration, and multimodal designs that remain reliable under missing modalities and real-world distribution shift.

## 1 Introduction

Triple-negative breast cancer (TNBC) is clinically defined by the absence of estrogen receptor (ER), progesterone receptor (PR), and human epidermal growth factor receptor 2 (HER2) expression, yet it encompasses multiple biological programs and microenvironmental states (1, 2). The resulting heterogeneity manifests in variable imaging appearance, diverse histomorphology, and distinct molecular drivers, which jointly limit the reliability of single-source biomarkers and motivate integrated, data-driven modeling (1, 3).

AI methods, spanning classical machine learning, deep learning, and modern foundation-model paradigms, have enabled large-scale feature learning from medical images, whole-slide pathology, and omics (4, 5). Prior TNBC-focused reviews have emphasized both the opportunity and the implementation barriers of AI in this disease context (6). In parallel, foundation models for vision have accelerated promptable and transferable representation learning, reshaping how segmentation and recognition pipelines can be built with reduced task-specific supervision (7). In TNBC, these methods are increasingly evaluated not only for discrimination (e.g., subtype classification), but also for clinically actionable prediction (e.g., response to neoadjuvant therapy, recurrence risk, and survival). However, translation remains constrained by study-design limitations, weak generalization across centers, and incomplete reporting of reliability and utility (8, 9).

This review provides a task- and modality-centric synthesis of AI for TNBC. Following the Methods section, Clinical context, data modalities, and AI foundations for TNBC summarizes the clinical context, data modalities, and AI foundations relevant to TNBC; Data and study-design challenges in TNBC discusses recurrent data and study-design limitations; the subsequent sections survey single-modality advances, multimodal fusion strategies, and clinically actionable prediction endpoints; and the final discussion distills cross-modal lessons, practical recommendations, and future directions.

## 2 Methods

### 2.1 Review design and scope

This article is a structured narrative Review rather than a systematic review or meta-analysis. Its purpose is to synthesize previously published research on AI for TNBC across radiology, digital pathology, genomics, and multimodal learning, while highlighting methodological advances, translational barriers, and clinically relevant research gaps. No unpublished data, personal communications, or original experimental results are introduced in this manuscript.

### 2.2 Literature selection principles

The evidence base was assembled through targeted searches of PubMed/MEDLINE, Web of Science Core Collection, Scopus, and Google Scholar, with the last update performed on 1 March 2026. Search terms combined TNBC-related descriptors (“triple-negative breast cancer”, “TNBC”, “basal-like breast cancer”) with AI and modality terms, including “machine learning”, “deep learning”, “radiomics”, “digital pathology”, “whole-slide image”, “genomics”, “transcriptomics”, “multi-omics”, “multimodal learning”, “treatment response”, “pathological complete response”, “survival”, and “prognosis”. Reference lists of relevant reviews and highly cited methodological papers were also screened to identify additional studies and reporting standards. Priority was given to studies that met one or more of the following criteria (i) evaluation in TNBC-specific or TNBC-enriched cohorts; (ii) focus on clinically meaningful tasks such as lesion segmentation, subtype classification, treatment-response prediction, recurrence-risk assessment, or survival modeling; (iii) inclusion of multimodal or multi-omics integration; and (iv) sufficient methodological detail to support critical appraisal of study design, validation, and interpretability. Studies using broader or mixed breast cancer cohorts were retained only when they provided methods, endpoints, or validation lessons directly relevant to TNBC, and they are identified as such in the narrative or tables where applicable.

### 2.3 Synthesis strategy

The literature was synthesized by modality and task, with explicit attention to study-design features that affect translational credibility, including patient-level data splitting, external validation, calibration, domain shift, missing-modality handling, and interpretability. To avoid over-comparing heterogeneous studies, reported performance values are interpreted together with cohort composition, endpoint definition, reference standard, validation level, and whether the evidence is TNBC-exclusive, TNBC-enriched, or extrapolated from broader breast cancer cohorts.

## 3 Clinical context, data modalities, and AI foundations for TNBC

### 3.1 Medical background

#### 3.1.1 Breast cancer in brief

Breast cancer remains the most frequently diagnosed malignancy among women worldwide and continues to impose a substantial public health burden. Contemporary clinical management relies on integrated assessment of imaging, pathology, and molecular markers. In routine practice, immunohistochemistry (IHC)-based surrogates (ER, PR, HER2, and Ki-67) are commonly used to stratify breast cancer into luminal, HER2-enriched, and triple-negative groups, which inform treatment selection and prognosis. Despite steady reductions in mortality in many regions driven by screening and therapeutic advances, outcomes remain heterogeneous across subtypes and clinical settings, motivating risk-adapted decision-making and more precise biomarkers (10–14).

#### 3.1.2 TNBC

TNBC is clinically defined by the absence of estrogen receptor (ER), progesterone receptor (PR), and HER2 expression and accounts for ∼10–15% of breast cancers, yet this operational definition collapses multiple tumor-intrinsic programs and microenvironmental states into a single category (1, 13). Transcriptomic taxonomies make this heterogeneity explicit Lehmann et al. initially described six TNBC subtypes (Basallike 1 (BL1), Basal-like 2 (BL2), Immunomodulatory (IM), Mesenchymal (M), Mesenchymal stem–like (MSL), and Luminal Androgen Receptor (LAR)) (15), and later refined this framework into four more tumor-intrinsic classes (BL1, BL2, M, and LAR) after evidence that IM and MSL signals were largely driven by immune and stromal admixture (16). Conceptually, BL1/BL2 phenotypes are enriched for proliferative and DNA-damage response programs, mesenchymal-like states emphasize epithelial-to-mesenchymal transition and stromal crosstalk, and LAR tumors exhibit androgen receptor signaling coupled to luminal differentiation. These programs can coexist within a tumor and shift under treatment pressure, producing multiscale heterogeneity that is expressed as variable imaging phenotypes, divergent histomorphology, and discordant molecular readouts.

Clinically, this coupled tumor–microenvironment architecture helps explain why single-modality biomarkers often fail to generalize across cohorts and treatment contexts (17, 18). pCR to neoadjuvant therapy and benefit from immunotherapy are influenced not only by cancer-cell genotype, but also by the “immune set-point” of the tumor microenvironment, which is partially captured by tumor-infiltrating lymphocyte (TIL) burden and composition. For example, intratumoral CD4^+^/CD8^+^ balance has been associated with favorable outcomes in TNBC (19), and efforts to standardize quantitative, guideline-concordant TIL assessment using digital pathology are emerging (20). These biology-to-clinic couplings motivate AI frameworks that integrate macroscopic imaging, cellular architecture, and molecular programs to model TNBC as an evolving system and to deliver more transportable predictions for diagnosis, risk stratification, and treatment response.

**Figure 1:**
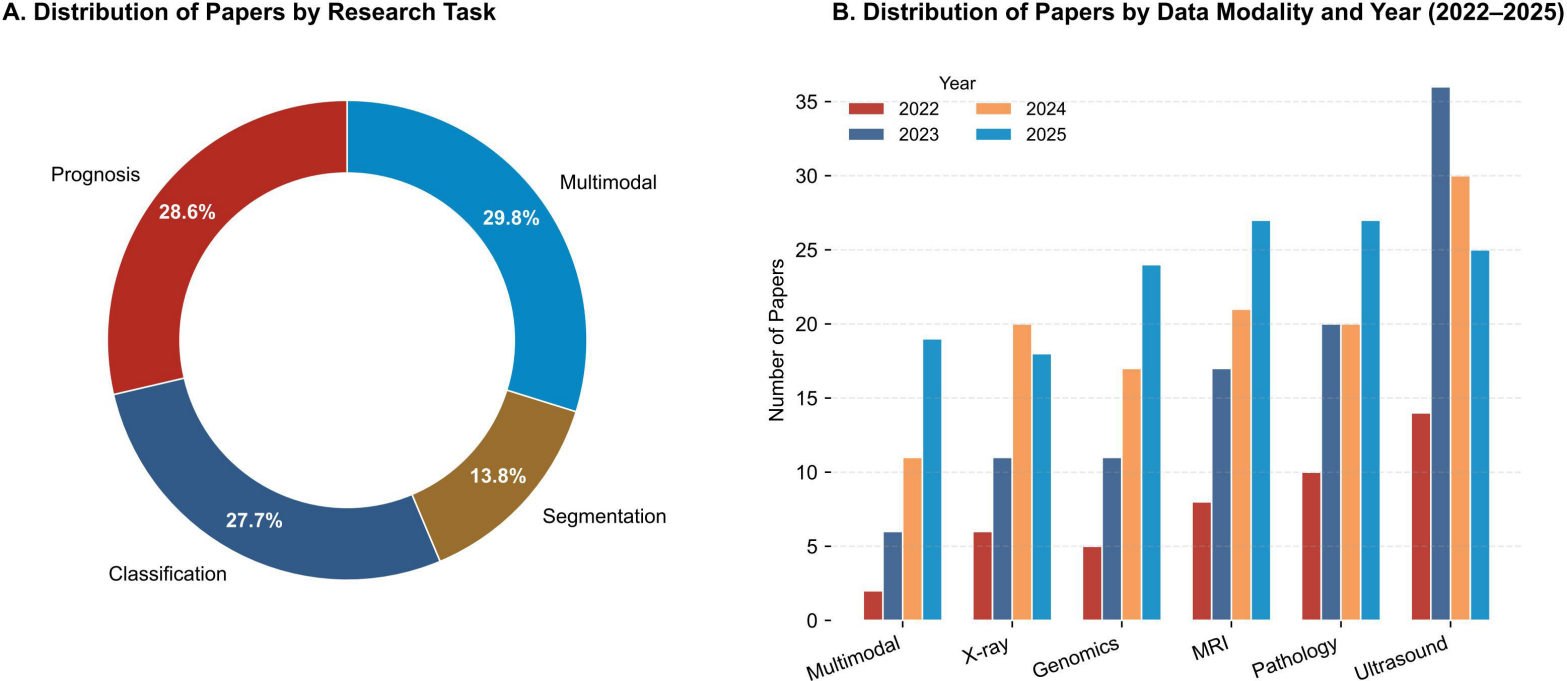
Distribution of studies on TNBC across research tasks (left) and data modalities (right). Counts were derived from manual coding of representative studies included in this narrative review. Literature was retrieved from PubMed/MEDLINE, Web of Science Core Collection, Scopus, and Google Scholar, with the last access on 1 March2026.Eachstudy was assigned to its dominant task and dominant data modality for descriptive purposes. The literature is dominated by single-modality imaging studies, while multi-omics and truly multimodal fusion remain relatively underrepresented. **(A)** Distribution of papers by research task. **(B)** Distribution of papers by data modality and year (2022–2025).

To provide a coarse landscape view, we categorize representative studies by primary task (e.g., segmentation, classification, response/survival prediction) and by data modality (radiology, pathology, genomics, and multimodal integration), as summarized in **Figure 1**. The counts were generated from the studies included in this narrative review after targeted searches of PubMed/MEDLINE, Web of Science Core Collection, Scopus, and Google Scholar, with the last access on 1 March 2026. This descriptive overview is intended to orient the reader rather than to support quantitative performance comparison across heterogeneous cohorts and evaluation protocols.

#### 3.1.3 Imaging data

Mammography is currently the most widely used diagnostic test in the world, which is a low-dose X-ray technique used for breast imaging that can reveal abnormalities such as masses, calcifications, and architectural distortion. This technique has advantages in early detection, has been proven to effectively reduce breast cancer mortality (21, 22), and is widely used for the early detection, early diagnosis, and reduction of mortality in breast cancer (23). In the past two decades, with the development of AI, the combination of mammography and AI has been used for the detection and prediction of breast cancer, and relevant research and applications have been increasing year by year, with future development continuing in this direction (24, 25). Earlier breast imaging CAD pipelines explored classical machine-learning for mass localization and rule-based or fuzzy-logic classifiers for mammographic tissue characterization, providing a methodological prehistory to modern deep-learning systems (26–29).

Ultrasound imaging (US) has become an important component of TNBC screening and diagnosis processes due to its lack of ionizing radiation, suitability for repeated bedside examinations, and friendliness toward dense breasts. For many years, breast ultrasound has been primarily used to differentiate cystic and solid lesions and has become an indispensable part of diagnosing breast abnormalities. In recent years, due to continuous technological innovation and application, ultrasound imaging, despite certain drawbacks, still serves as a rapid tool (30). The high accessibility and relatively low cost of ultrasound equipment make it particularly attractive and important for breast cancer screening and diagnosis in resource-limited countries (31). Furthermore, ultrasound elastography has achieved significant progress in recent years. Combining advanced elastography methods with AI and standardized protocols is expected to establish ultrasound elastography as an important tool for early breast cancer detection, potentially improving patient prognosis through early intervention (32, 33).

Magnetic Resonance Imaging (MRI) plays an increasingly important role in the detection, staging, and efficacy evaluation of breast cancer due to its excellent soft tissue contrast and multiparametric imaging capabilities. MRI is more sensitive in capturing changes in tumor hemodynamics, tissue structural features, and molecular-level metabolic information, offering a significant advantage, particularly in patients with dense breast tissue. Mammography, as a general method, has a relatively high false-negative rate for invasive breast carcinoma (IBC). In contrast, MRI is the most sensitive method for detecting invasive carcinoma, comparable to mammography for detecting ductal carcinoma *in situ* (DCIS), making the two complementary. Therefore, MRI can serve as a supplement to mammography, effectively addressing its limitations in dense breasts and high-risk populations (34–36). Dynamic Contrast-Enhanced MRI (DCE-MRI), by reflecting tumor blood flow and permeability, provides functional information on tumor angiogenesis and invasiveness; sequences such as diffusion-weighted imaging (DWI) supplement quantitative metrics of cell density and tissue microstructure. These two modalities constitute the core value of Multiparametric Magnetic Resonance Imaging (mpMRI) in molecular subtyping, prognosis, and drug response assessment. For TNBC, DCE-MRI, DWI, and multiparametric analysis can provide crucial information regarding tumor biological behavior. As various methods continue to mature, MRI will be improved and hold more potential in the future (37, 38).

Pathological images, particularly Hematoxylin and Eosin (H&E)-stained tissue sections, are the “gold standard” for breast cancer diagnosis, offering a direct visualization of key pathological features such as tumor cell morphology, nuclear atypia, and TILs. Traditional pathological diagnosis relies heavily on the pathologist’s subjective experience and faces challenges such as heavy workloads and inter-observer variability in diagnostic consistency (39). With the development of digital pathology, high-resolution whole-slide images (WSIs) have become standard (40), laying the foundation for the application of computational pathology and AI (41). AI models can perform quantitative analysis on WSIs containing millions or even billions of pixels, capturing micro-features that are difficult for the human eye to discern. For example, the precise quantification of TILs abundance is of great value in assessing the pCR rate to neoadjuvant chemotherapy (NAC) in TNBC patients. Specifically in TNBC, AI based on pathological images can quantitatively evaluate TILs abundance, which is highly correlated with the pCR rate (42), providing a basis for predicting the benefit of immunotherapy. The applications of AI in pathological images mainly include aiding diagnosis, prognosis prediction, and biomarker discovery, offering new avenues for a deeper understanding of the biological behavior of TNBC (42).

#### 3.1.4 Genomics

Genomics is a core field in life science and medical research, aiming to systematically analyze and understand the structure, function, and dynamic regulatory processes of an organism’s entire genome. The completion of the Human Genome Project (2003) ushered in a new era of defining diseases at the molecular level, driving the transition of medicine from a “one-size-fits-all” approach to individualized medicine (43, 44). Early applications of genomic medicine primarily focused on disease risk assessment, disease diagnosis and classification, and targeted therapy and clinical trials (45). In recent years, with the rapid development of high-throughput sequencing (HTS) technology and Next-Generation Sequencing (NGS), the applications of genomics have expanded to areas such as cancer pharmacogenomics, rare disease diagnosis, infectious disease outbreak tracking, non-invasive prenatal testing and newborn screening, pharmacogenomics, and drug repurposing, playing a critical role in the advancement of precision medicine (43, 46, 47). The application of genomics is particularly prominent in oncology. Through methods such as whole-genome sequencing (WGS), whole-exome sequencing (WES), and RNA sequencing (RNA-seq), comprehensive molecular analysis of tumors can be performed to precisely identify gene variations driving tumor growth, thereby guiding patients in selecting the most effective targeted or immunotherapies (48). TNBC, as a molecularly highly heterogeneous breast cancer subtype, poses a challenge to traditional diagnosis and treatment. Through NGS technology, researchers have discovered various recurrent pathogenic mutations in TNBC (such as tumor protein p53 [*TP53*] mutations and aberrations in the phosphatidylinositol4,5-bisphosphate 3-kinase catalytic subunit alpha [*PIK3CA*] and DNA repair pathways) (49). Specifically, a strong association exists between TNBC and Homologous Recombination Deficiency (HRD). Relevant studies have developed Machine Learning (ML) classifiers based on gene expression to identify the HRD status, providing an alternative approach for personalized treatment with Poly (ADP-ribose) polymerase (PARP) inhibitors or platinum-based chemotherapy (50). Furthermore, comprehensive genomic and transcriptomics analysis has revealed the low tumor mutation burden of TNBC and its association with an immunosuppressive Tumor Microenvironment (TME) (51). DNA methylation studies, meanwhile, have subtyped TNBC into epigenetic subtypes associated with different prognoses and immune evasion strategies. These in-depth molecular insights are providing important directions for the precise diagnosis, prognosis prediction, and novel targeted therapies (such as immune checkpoint therapies) for TNBC (52).

### 3.2 Foundations of artificial intelligence technology in the medical field

#### 3.2.1 Machine learning

Machine learning is used in TNBC research to learn reproducible patterns from imaging, pathology, molecular profiles, and clinical variables (53). The clinically relevant tasks are not algorithm categories in isolation, but diagnosis, subtype inference, response prediction, recurrence-risk stratification, and survival modeling (54–56). Across these tasks, simpler models such as regularized regression, random forests, support vector machines, and gradient boosting remain useful baselines, particularly for small molecular cohorts where high-dimensional overfitting is a persistent risk (59, 61). Their value depends on transparent feature selection, patient-level splitting, external testing, and calibration rather than headline discrimination alone.

#### 3.2.2 Deep learning

Deep learning is most useful when the input is high-dimensional and weakly structured, such as MRI volumes, ultrasound videos, mammography views, and whole-slide pathology images. In TNBC, the main contribution of deep learning is automated representation learning for lesion delineation, weakly supervised WSI prediction, longitudinal response modeling, and multimodal fusion (57, 64, 65). However, the maturity of these applications differs. Image-based response prediction and computational pathology have begun to show externally tested signals, whereas many subtype-classification and omics-fusion studies remain retrospective and internally validated. Thus, architectural novelty should be interpreted together with data quality, endpoint relevance, and validation design.

#### 3.2.3 Representative segmentation model families

**Figure 2:**
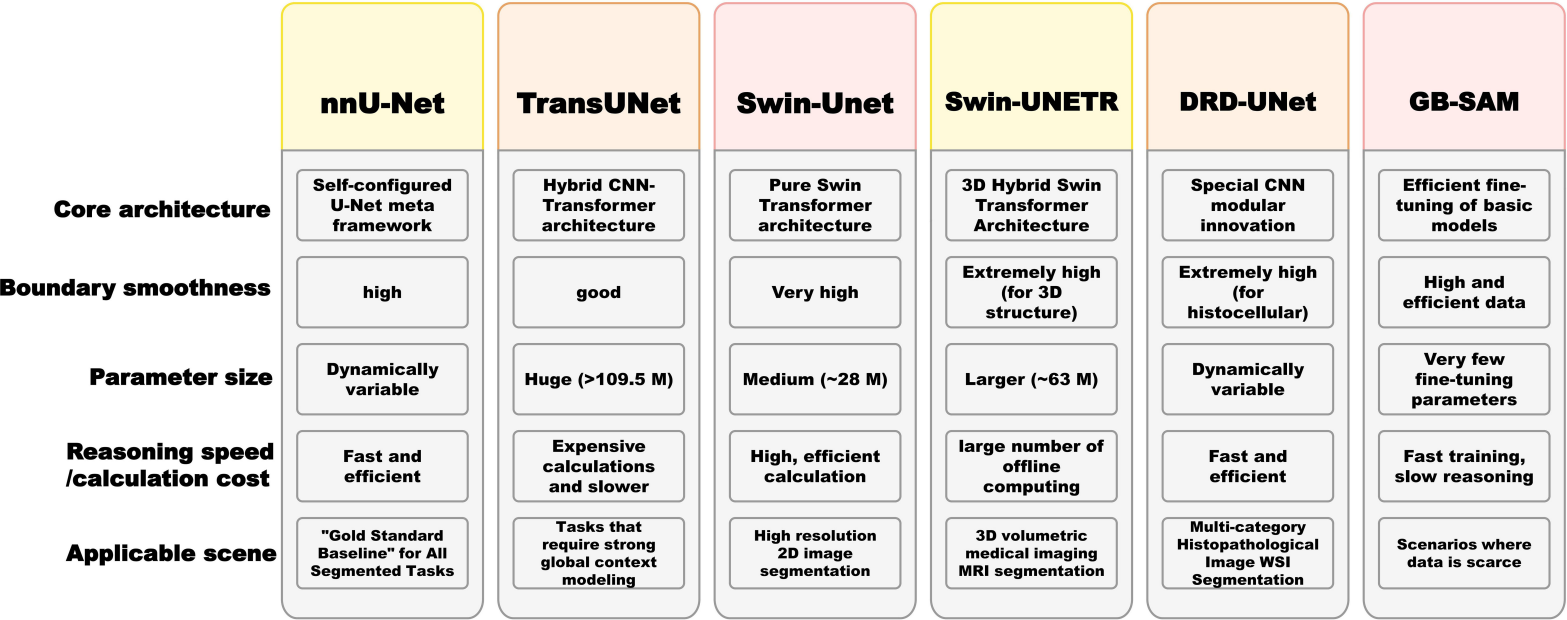
Comparative analysis of representative medical image segmentation architectures. The figure contrasts models (nnU-Net, TransUNet, Swin-Unet, Swin-UNETR, DRD-UNet, and GB-SAM) in terms of architectural bias, boundary fidelity, model size, computational efficiency, and typical best-use scenarios. U-Net-derived baselines remain strong on limited data, whereas transformer/foundation style approaches primarily add value when global context or weak/interactive supervision is needed.

**Figure 2** summarizes representative segmentation model families without treating architecture as a proxy for clinical readiness. U-Net-derived baselines and nnU-Net-style self-configuring pipelines remain strong practical references when labeled data are limited and protocols are well controlled (66). Transformer and hybrid CNN–Transformer designs, including TransUNet, Swin-Unet, and Swin-UNETR, can improve long-range context modeling, but their benefit in TNBC depends on cohort size, input resolution, and whether external testing is performed (67–70). Other U-Net variants developed in broader medical-imaging settings, such as DRD U-Net, are useful as methodological references but should not be read as TNBC-specific evidence without disease-specific validation (71).Foundation-model approaches such as SAM may reduce annotation burden, yet zero-shot medical performance remains unstable and usually requires task-specific adaptation (7, 74, 75). For TNBC studies, the main clinical question is therefore not which backbone is newest, but whether segmentation improves downstream response or prognosis prediction under leakage-free, patient-level evaluation.

#### 3.2.4 Representative classification model families

Classification tasks include benign–malignant differentiation, TNBC versus non-TNBC classification, molecular subtype inference, and biomarker prediction.

Evidence is strongest when the task is linked to an actionable endpoint and tested outside the training institution. Weakly supervised WSI methods such as transMIL and CLAM are useful because they learn from slide-level labels and can provide attention-based localization cues (77, 78).Multimodal models that combine radiology, pathology, and structured clinical variables may better capture TNBC heterogeneity, but reported gains should be interpreted cautiously when cohorts are mixed, endpoints differ, or external validation is absent (57, 79). In practice, classification evidence is most convincing when performance is reported with sensitivity, specificity, calibration, subgroup behavior, and failure modes rather than accuracy alone.

#### 3.2.5 Representative prognostic model families

Prognosis prediction in TNB centers on pCR, disease-free survival, recurrence, and overall survival. The most clinically interpretable approaches are those that align model inputs with decision windows, such as baseline or early-treatment MRI for neoadjuvant response, pretreatment pathology for immune context, and longitudinal or multimodal features for recurrence risk. TNBC-specific studies, including baseline multiparametric MRI response models, provide direct disease-relevant evidence but often show more modest performance under external testing than under internal splits (65). Broader breast cancer studies can still be informative for architecture or workflow design, yet their performance should not be treated as TNBC-specific unless subgroup validation is reported (80). Graph-based TME models and immune-structure models are promising because they encode spatial Relationships among tumor, stromal, and immune compartments rather than only aggregate features (81, 82). Population-level survival models based on SEER or similar registries can support risk stratification, but they remain sensitive to registry bias, treatment heterogeneity, and missing molecular information (65). Mechanism-informed frameworks such as ARIADNE add biological interpretability, although their clinical use requires independent validation and experimental corroboration of inferred state transitions (83).

#### 3.2.6 Quantitative indicators for model performance evaluation

Because TNBC datasets are often imbalanced and endpoints are heterogeneous, no single metric is sufficient. For classification, accuracy should be accompanied by sensitivity, specificity, precision, F1score, and ROC-AUC or precision-recall AUC where class imbalance is substantial. For segmentation, Dice and IoU summarize overlap, whereas HD95 or related distance measure describe boundary errors that may affect radiomics features. For survival and recurrence modeling, C-index time-dependent AUC, hazard ratios, confidence intervals, calibration, and decision-curve analysis are more informative than discrimination alone. Throughout this review, performance values are interpreted as study-specific summaries rather than directly comparable rankings, because cohorts differ in TNBC definition, endpoint timing, sample size, image acquisition, sequencing platform, and validation protocol.

#### 3.2.7 Integrated multimodal workflow

**Figure 3:**
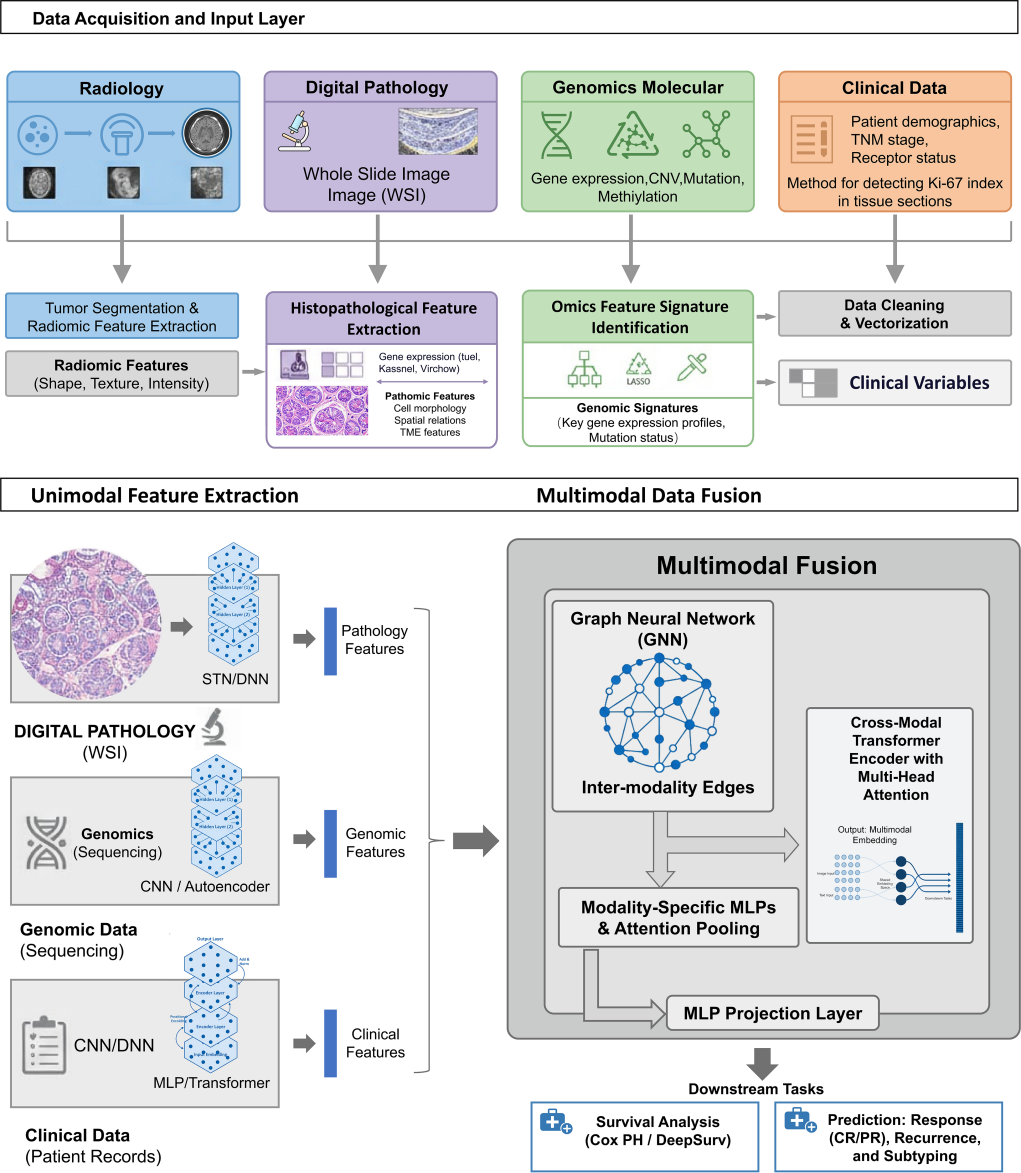
End-to-end multimodal AI workflow for TNBC from data acquisition to downstream clinical endpoints. Radiology, digital pathology WSI, and genomics/molecular profiles are processed by modality-specific feature extractors and fused (e.g., via GNNs or cross-modal Transformers) to support subtype inference, treatment response prediction, and survival modeling. Fusion is most effective when modalities contribute complementary signals, but depends on robust alignment, missing-modality handling, and external validation.

TNBC, due to its high heterogeneity and aggressiveness, poses severe challenges to clinical prediction models (1). To address this challenge, MML models integrating multi-source heterogeneous data have become a core strategy (84). As shown in **Figure 3**, a comprehensive workflow begins with data acquisition radiological images, pathological WSI, genomics, and clinical information (85). Subsequently, specialized DL models extract modality-specific features—such as radiomics, cellular architecture, and molecular profiles—which are then integrated in the multimodal fusion stage (86). While advanced techniques like GNNs or cross-modal Transformers generate unified representations to support survival analysis and response prediction (79, 84), it must be emphasized that the practical bottleneck of multimodal fusion often lies not in algorithmic complexity, but in the system’s capacity to handle “missing modalities” effectively. The clinical robustness of such frameworks depends more on managing incomplete data common in oncology practice than on the fusion architecture itself. This information-rich fused representation finally provides critical decision support for TNBC precision medicine (79).

## 4 Data and study-design challenges in TNBC

**Figure 4:**
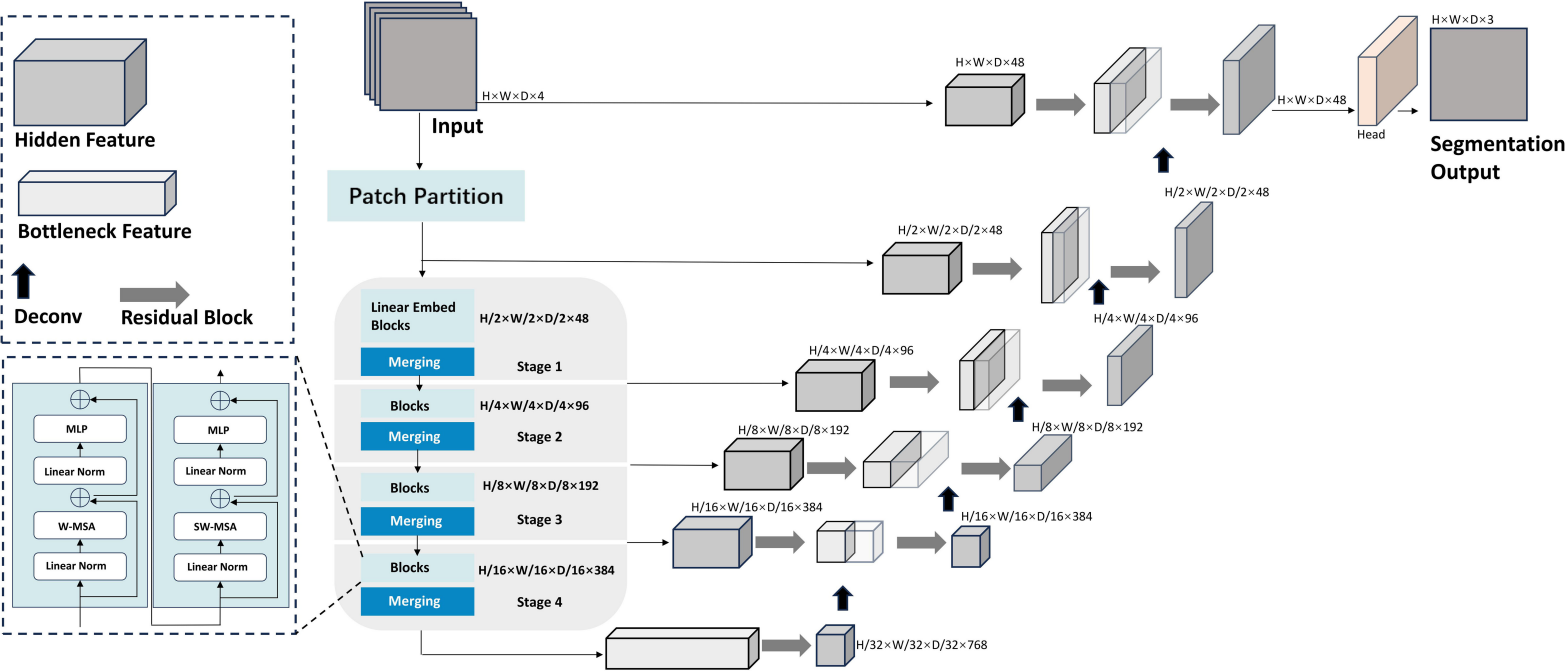
Schematic of the Swin UNETR architecture. A Swin Transformer encoder with hierarchical patch merging provides multi-scale context, and a convolutional decoder with skip connections restores spatial detail. Hybrid Transformer–CNN designs aim to combine long-range context with precise boundary localization.

**Figure 5:**
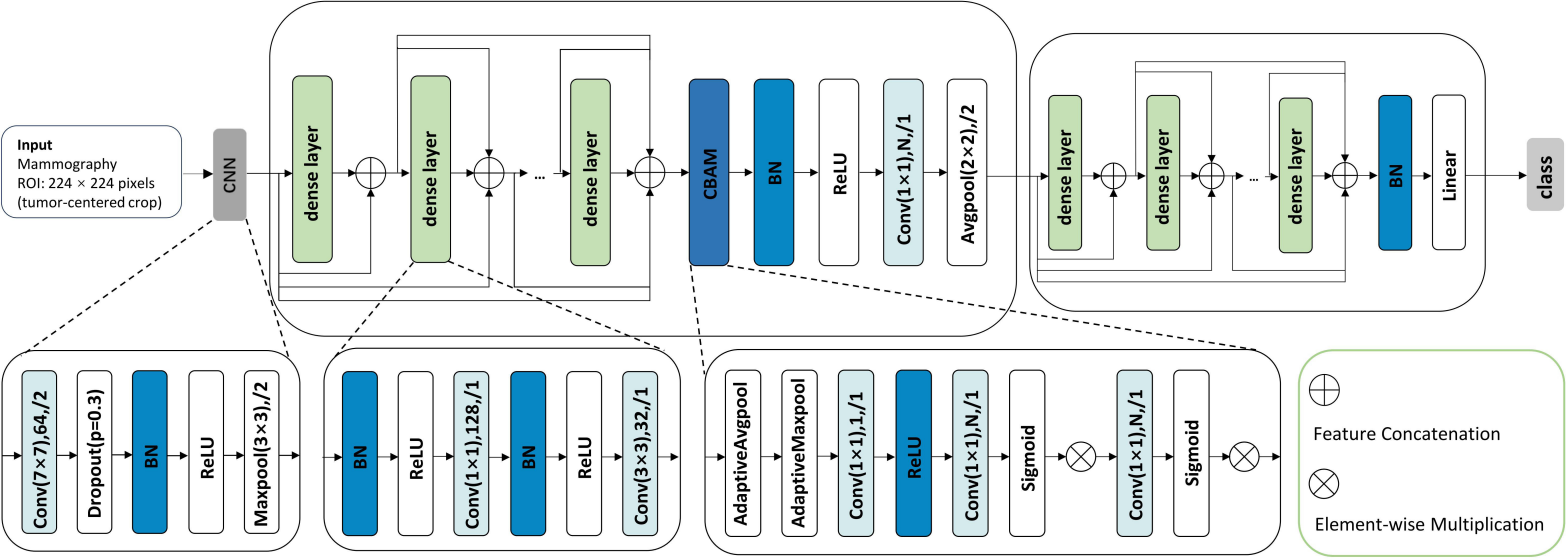
DenseNet121-CBAM pipeline for mammography ROI classification. DenseNet121 extracts hierarchical features via dense connectivity, while CBAM refines representations using channel and spatial attention before the final classifier. Attention can improve lesion-focused representations, but gains depend on ROI quality and careful leakage avoidance.

AI studies in TNBC are frequently constrained by small and non-representative cohorts, imperfect outcome definitions, and substantial heterogeneity in acquisition protocols (scanner/vendor, imaging parameters, staining and slide preparation, sequencing platforms, and site-specific clinical pathways). These factors introduce confounding and distribution shift, complicate cross-study comparisons, and inflate the risk of optimistic bias if patient-level splits, external validation, and calibration are not handled rigorously. This section synthesizes the most recurrent data- and design-level pitfalls reported across modalities, with a focus on class imbalance, weak or noisy supervision, and multi-center generalization. **Figures 4** and **5** illustrate representative model structures related to these data-design challenges. **Figure 4** presents the Swin UNETR-style hybrid Transformer-CNN segmentation framework, whereas **Figure 5** presents the DenseNet121-CBAM mammography ROI-classification workflow.

The data imbalance problem significantly affects the performance and generalization ability of models in AI research applied to TNBC, especially in medical imaging modalities such as X-ray, MRI, and ultrasound. Due to the scarcity of TNBC cases compared to other breast cancer subtypes, datasets often exhibit class distribution bias, which leads to a reduction in recall (SEN) for the minority class.

In X-ray imaging (such as Contrast-Enhanced Mammography (CEM)), the detection of TNBC faces challenges due to unequal sample sizes. Ma et al. used the Synthetic Minority Over-sampling Technique (SMOTE) to generate synthetic samples, balancing the proportion of positive and negative cases and preventing the model from being biased toward the majority class (e.g., benign or non-TNBC tumors); their results showed an increase in overall ACC to approximately 85% (87).

Turning to the MRI modality, the prediction of axillary lymph node metastasis in TNBC is often constrained by the uneven distribution of metastatic and non-metastatic samples. A small cohort study revealed that metastatic samples accounted for only 26% of the total. Shen et al. confirmed that by combining clinical and Radiomics features and setting class weights (class_weight=“balanced”) in a random forest classifier to compensate for minority class loss, this strategy increased the AUC to 0.89. Although the bias was not completely eliminated, the SEN was improved in more clinically realistic evaluation settings (88).

In ultrasound imaging, the automatic prediction system for TNBC faces the problem of insufficient minority class samples. Datasets are often dominated by non-TNBC cases, leading to an initial model SEN of only 71%. Boulenger et al. adopted a strategy of undersampling the majority class (non-TNBC tumor samples) combined with dynamically adjusting the class ratio during each training epoch, which improved model performance to a SEN of 81%. Their study showed that incorporating k-fold stratified cross-validation ensured that the class ratio in each fold was consistent with the overall dataset, partially mitigating bias and improving generalization ability (89).

Switching to the pathological image modality, data imbalance is particularly prominent in the histological feature analysis of TNBC, due to the insufficient representation of histological categories (such as microcalcification or muscle tissue) involved in NAC response prediction. The small sample size leads to uneven class distribution among patient slides. Fisher et al. employed the RUSBoost (Random UnderSampling Boosting) ensemble tree method, combining random undersampling and boosting algorithms to prioritize the identification of minority class features, which improved the balanced ACC of response prediction from an initial 65% to 78%. The study also indicated that this method effectively reduces information loss when dealing with inter-institutional slide quality variation (62).

In the broader histopathological classification, the molecular subtype classification of TNBC faces severe under-representation of the HER2 and TNBC categories; datasets show the TNBC proportion is less than 20%. To address this, Jang et al. employed a Weakly Supervised Learning (WSL) framework, merging multiple datasets (such as TCGA and Korea University Guro Hospital (KG)) to augment the bag of minority positive samples, which boosted the Area Under the Receiver Operating Characteristic Curve (AUROC) for the HER2 and TNBC classes to 0.749. This merging strategy significantly alleviated the label imbalance problem and improved the model’s recognition performance for minority subtypes (90).

Furthermore, in recurrence prediction, histopathological image datasets showed that recurrent samples accounted for only 5%. After using SMOTE to generate synthetic minority class samples, the training set was balanced to a near 1–1 ratio of recurrent to non-recurrent cases, boosting the AUC to 0.82. Sahoo et al. applied this method to an HER2 positive cohort, but the principle is equally applicable to similar imbalance scenarios in TNBC cases (91). When using the Conditional Denoising Diffusion Probabilistic Model (CDDPM) for fusing multimodal data to process histological images, the under-representation of the TNBC-related necrosis category led to a decrease in segmentation ACC. Akbari et al. further improved the PREC of the minority class (e.g., necrosis) by approximately 10% by employing strategic patch creation to ensure balanced representation of all tissue categories and introducing a Class-Balanced Loss Function (CBLF) (92).

In the genomics field, transcriptomics and methylation datasets for TNBC can be highly imbalanced (e.g., TNBC samples may comprise only 16% of the cohort), which can cause ML models to overlook critical genetic differences.

Kothari et al. prioritized the F1-score over ACC to evaluate Decision Tree (DT) and random Forest (RF) models, identifying 20 differentially expressed genes (15 downregulated, 5 upregulated) on TCGA data, and mitigated randomness through repeated train–test splits, ensuring balanced performance under the 84–16 bias (59).

Advanced resampling can further reduce false negatives under severe imbalance. For example, integrating SMOTEENN with RF on breast cancer genomics datasets (imbalance ratios up to 18%) can boost average performance to 94.49% and reduce FN rates. Gurcan and Soylu applied this strategy to prognosis prediction, which is especially suitable for minority outcomes such as TNBC recurrence (93).

Beyond resampling, transcriptomics studies increasingly report imbalance-aware metrics (e.g., Balanced Accuracy (BACC) and Matthews Correlation Coefficient (MCC)) and use high-dimensional feature control (e.g., Recursive Feature Elimination (RFE) and Elastic Net (EN)) to stabilize learning, leading Saadh et al. to achieve an AUC of 0.92 using such methods (94).

Finally, when multimodal methods integrate imaging, pathology, and genomics, the Data Imbalance Problem (DIP) of TNBC can be amplified, because the accumulation of biases from each modality may cause fusion models to favor the majority class.

For instance, in neoadjuvant chemotherapy (NAC) response prediction where pCR and non-pCR samples are imbalanced, a Class-Weighted Binary Cross-Entropy Loss Function (CWBCE Loss) can set higher weights for the minority class to compensate for misclassification penalties, thereby boosting ACC to 90%. Khan et al. validated this strategy using an Attention-based Multiple Instance Learning (Attn-MIL) framework when processing pre-processed histological images (95).

In fully automated pipeline systems affected by DIP, the Precision–Recall Curve (PRC) is often preferred for evaluation, with Mao et al. reporting an AUPRC of 0.833. They alleviated bias by fusing multi-center data through a fully automated pipeline with multimodal integration (96).

When integrating histopathology and ultrasound images, an explainable AI framework can also handle imbalance by computing inverse-frequency class weights (e.g., normal class weight reached 1.977) to improve the F1-score. Alom et al. demonstrated applicability for TNBC detection using an Explainable AI-Driven Deep Neural Network (DNN), while noting that minority-class scarcity remains a limitation that may require oversampling or ensemble strategies (97).

## 5 Single-modality AI for TNBC

This section synthesizes AI methods for TNBC within individual data modalities, emphasizing (i) clinically grounded tasks (lesion delineation, molecular subtyping, response and prognosis prediction), (ii) methodological trends (representation learning, longitudinal modeling, weak supervision, foundation models), and (iii) evidence quality (external validation, multi-center robustness, and reproducibility). Throughout, reported performance should be interpreted in light of cohort composition, endpoint definition, and evaluation protocol heterogeneity.

### 5.1 MRI analysis

Breast MRI offers high sensitivity and rich functional information, particularly through dynamic contrast-enhanced MRI (DCE-MRI) and, when available, diffusion-weighted imaging (DWI) and derived parametric maps (34). Representative MRI-based TNBC AI studies spanning segmentation, subtyping, treatment response, and prognosis are summarized in **Table 1**, including cohort size, endpoint definitions, validation design, and key results. Accordingly, MRI has become a primary substrate for AI-driven TNBC analysis, spanning three recurring problem classes (i) tumor segmentation as a prerequisite for quantitative analysis, (ii) phenotype inference and outcome prediction (e.g., subtype, pCR, recurrence), and (iii) integrated or multi-task frameworks that align with computer-aided diagnosis (CAD) workflows (98).

**Table 1:** Representative MRI-based AI studies relevant to TNBC, spanning segmentation, subtyping, treatment response, and prognosis.

Study	N (Dataset)	Input	Task/Endpoint	Model	Split & CV	Patient-level split	External validation	Endpoint definition	Key results
Xu(2023) (106)	301	DCE-MRI	Segmentation/tumor mask	nnU-Net	5 1 (T/Ts); 5-fold	Yes	No^a^	Manual expert contour	Seg Dice=0.93
Xu (2025) (102)	282	DCE-MRI + DWI	Response prediction/pCR	3D ResNet + S-transformer	197 85 (T/Ts); none	Yes	No	Miller-Payne grade/pCR	Cls AUC = 0.76
Wu (2022) (107)	56	DCE-MRI + DWI	Response prediction/pCR	DL + MBI	45 11 (T/Ts); none	Yes	No	Miller-Payne (No inv. tumor)	Cls AUC = 0.89
Tran (2025) (108)	169	DCE-MRI	Subtype prediction/subtype	Radiomics + LR	135 34 (T/Ts); 5-fold	Yes	No	IHC/FISH (St. Gallen)	Cls AUC = 0.84
Cai(2025) (109)	1353	mpMRI	TNBC classification/ TNBC	XGBoost	963 413 (T/Ts); 10-fold	Yes	No^b^	Pathological IHC	Cls AUC = 0.71
LoGullo (2024) (110)	541	MRI (FGT)	TNBC classification/ TNBC	Radiomics + SVM	250 291 (T/Ts); none	Yes	No	IHC (ER/PR/HER2-)	Cls AUC = 0.71
Guo (2022) (111)	272	DCE-MRI	Seg + Cls/mask + TNBC	CNN-SVM	190 82 (T/Ts); 5-fold	Yes	No	Dice coeff./IHC status	Seg Dice=0.93; Cls Acc=0.93

FGT, fibroglandular tissue; LR, logistic regression; T, training set; Ts, test set; CV, cross-validation; Cls, classification; Seg, segmentation; MBI, molecular breast imaging; FISH, fluorescence *in situ* hybridization.

Reported performance is strongly influenced by cohort size, endpoint definition, and validation strategy, highlighting the need for external testing and standardized reporting.

#### 5.1.1 Segmentation

Accurate tumor segmentation is foundational for downstream radiomics and model-based phenotype characterization (99). U-Net-type encoder–decoder architectures remain dominant due to their multiscale feature fusion via skip connections (100). In TNBC-related MRI studies, 3D implementations are frequently preferred to exploit volumetric context. For example, Gao et al. reported that a self-configured nnU-Net-style pipeline on longitudinal DCE-MRI achieved a Dice score of 0.860 on an independent test set (101). Nevertheless, convolutional designs can be limited in modeling long-range interactions when tumor morphology is highly variable, motivating hybrid CNN–Transformer encoders that provide global context. Architectures such as Swin UNETR exemplify this direction by coupling a Swin Transformer encoder with a convolutional decoder for spatial detail recovery (68, 70). While such hybrids have shown strong performance across multiple 3D medical segmentation benchmarks, their application to TNBC is nascent. Recent studies, such as the S-transformer approach by Xu et al. (102), have begun to demonstrate their utility, yet the broader evidence base remains comparatively limited and would benefit from dedicated multi-center benchmarking under harmonized protocols.

#### 5.1.2 Reducing annotation burden

Supervised segmentation depends on dense, expert annotations, which are costly and often infeasible at scale (4). Two practical directions are increasingly explored. First, self-supervised pretraining, foundation model transfer, and parameter-efficient adaptation can improve data efficiency when TNBC-specific labels are limited (72, 73). Second, *promptable* segmentation pipelines combine weak localization (e.g., coarse boxes from an object detector) with a general-purpose segmentation foundation model (e.g., SAM) to produce pixel-level masks with substantially reduced manual effort (7, 74). Automated hybrids such as nnSAM further illustrate how SAM-style prompting can be combined with nnU-Net-like medical segmentation priors (76). For TNBC MRI, a key open question is how reliably such prompt-driven approaches generalize across scanners, acquisition protocols, and enhancement kinetics without task-specific adaptation.

#### 5.1.3 Classification and prediction

**Figure 6:**
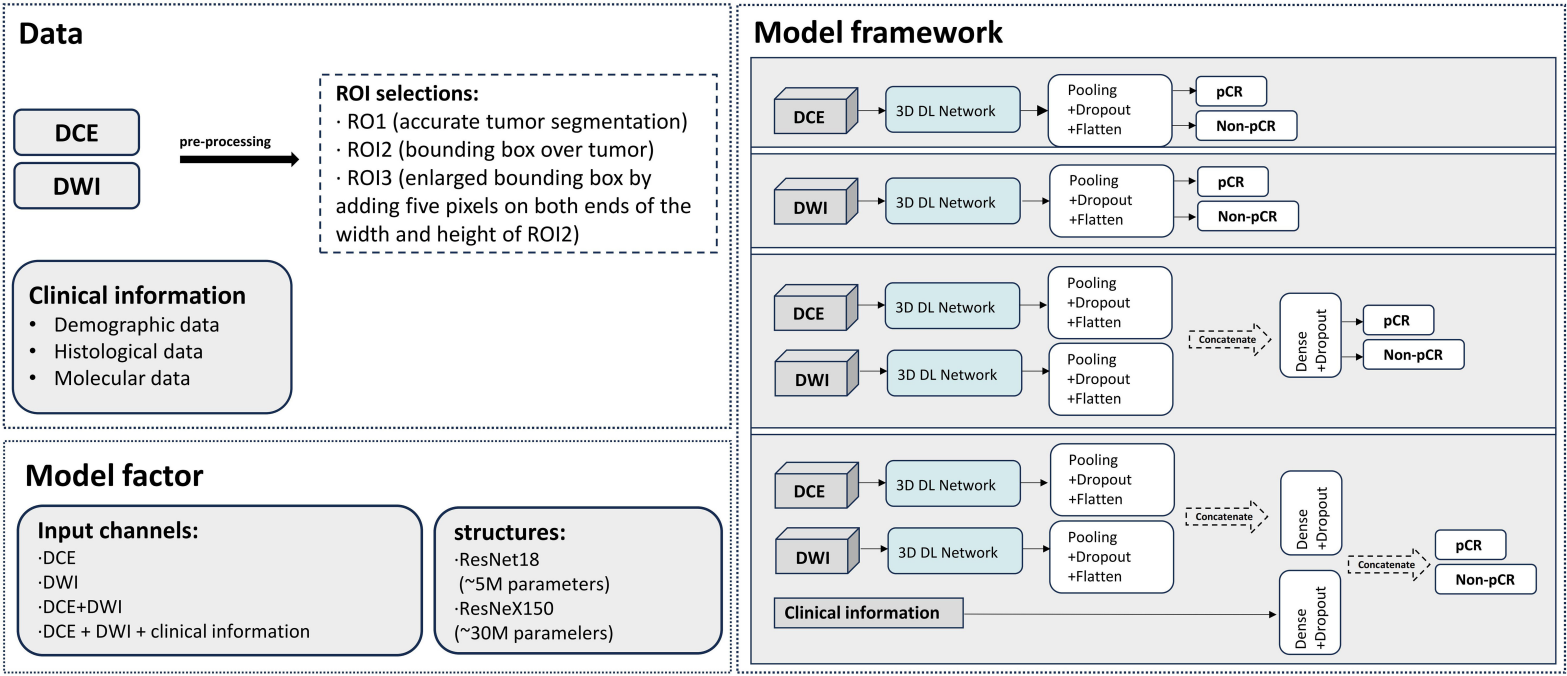
Framework of a 3D deep-learning pipeline for NAT response prediction in TNBC. Illustrates baseline DCE/DWI and clinical inputs, ROI specification (segmentation vs. bounding box), feature fusion, and pCR/non-pCR prediction (65). ROI definition and fusion design are recurring sources of performance variation; leakage-free splits and calibration are essential before clinical use.

Beyond detection, MRI-based AI targets clinically consequential endpoints molecular subtype inference, treatment response prediction, and recurrence risk stratification. Most studies leverage DCE-MRI due to its hemodynamic information; mp-MRI integration (e.g., DCE-MRI + DWI) is a consistent theme (101). For neoadjuvant chemotherapy (NAC) response, longitudinal modeling is particularly compelling approaches that exploit *on-treatment* changes often outperform those using only baseline scans, aligning with the clinical intuition that early tumor dynamics encode chemosensitivity (103). **Figure 6** summarizes a representative 3D MRI-based NAT response-prediction workflow, linking DCE/DWI inputs, ROI specification, clinical variables, feature fusion, and pCR/non-pCR classification. Huang et al. reported a deep model integrating baseline and mid-treatment mp-MRI with an AUC of up to 0.958 in independent blinded testing (103). Such results support the promise of “delta”-style representations (34, 99), but their transferability hinges on consistent timepoint definitions, robust handling of missing sequences, and careful avoidance of information leakage from post-treatment correlates.

#### 5.1.4 Feature fusion and uncertainty

Performance gains commonly arise from fusing complementary representations (radiomics + deep features; imaging + clinico-pathological variables). For instance, integrating multimodal MRI deep features with variables such as pTNM staging and Ki-67 has been reported to improve recurrence risk stratification in multi-center cohorts (80). Ensemble frameworks are also used to stabilize predictions and quantify uncertainty, though calibration and decision-analytic evaluation remain underreported in much of the literature (65).

#### 5.1.5 Multi-task learning and workflow alignment

MTL aims to unify segmentation and downstream prediction within a single framework to better match clinical CAD pipelines (104, 105). A common design is segmentation-first followed by outcome modeling from the segmented region, either as a pipeline or via joint optimization (80, 96). For clinical credibility, MTL studies should explicitly report whether segmentation is trained and evaluated on the same distribution as prediction, how errors propagate, and whether end-to-end training yields measurable benefit beyond a well-tuned modular baseline.

### 5.2 Ultrasound image analysis

Ultrasound is widely available and cost-effective, making it central to breast lesion assessment and follow-up. Representative BUS studies for segmentation and classification/subtyping, with split strategy and validation details, are summarized in **Table 2**. AI research in TNBC-related ultrasound can be organized into three themes (i) lesion detection/segmentation and benign–malignant classification, (ii) multimodal and volumetric extensions (e.g., elastography, Doppler, Automated Breast Ultrasound System (ABUS)) to improve reproducibility and coverage, and (iii) integrated multi-task or end-to-end pipelines toward prognosis-aware clinical decision support (57, 112–114). However, ultrasound-specific challenges (operator dependence, acquisition heterogeneity, speckle noise, and boundary ambiguity) systematically limit robustness, particularly under external validation and across devices (114).

**Table 2:** Representative breast ultrasound (BUS) AI studies for TNBC, including segmentation and classification/subtyping tasks.

Study	N (Dataset)	Split (T/V/Ts)	Modality	Task	Model	Pt-level split	Ext. valid.	Endpoint definition	Key results
Cho (2022) (128)	943^a^	4 1 (Ts)	BUS	Seg + Cls	RFS-UNet	Yes	No	Mask Manual contour; Cls Pathological B/M	Dice 0.90
Luo (2022) (129)	1,994	10-fold CV	BUS	Seg + Cls	U-Net + ResNet	Yes	No	Tumor boundaries and histological B/M	AUC 0.95
Misra (2023) (124)	212	137 34 41 (Ts)	B-mode + SE	Seg + Cls	W-MM-UNet	Yes	No	BI-RADS criteria and pathological diagnosis	Dice 0.79
He (2023) (118)	943^a^	5-fold CV	BUS	Segmentation	HCTNet	Yes	No	Expert-annotated tumor masks	Dice 0.92
Islam (2024) (125)	943^a^	7 1.5 1.5	BUS	Seg + Cls	EDCNN	Yes	No	Pathology-confirmed B/M status	Acc 0.97
Abhisheka (2024 (130)	943^a^	5-fold CV	BUS	Classification	HBMD-Net	Yes	No	Histology-based B/M labels	Acc 0.99
Aslam (2025) (131)	943^a^	8 2 (Ts)	BUS	Segmentation	HANet	Yes	No	Radiologist-verified segmentations	Dice 0.92

B/M, benign/malignant; SE, strain elastography; HANet, hybrid attention net. [a] Combined cohort from BUSI and UDIAT datasets.

Robustness improves when studies report stratified splits, calibration, and cross-site validation in addition to headline accuracy metrics.

#### 5.2.1 Segmentation

Segmentation provides ROIs for radiomics and downstream classification while enabling measurement of clinically relevant morphology (e.g., size, irregularity) (89, 115, 116). U-Net variants remain the workhorse; improvements typically target better boundary delineation and context modeling through attention, CNN–Transformer hybrids, dual-path designs, or explicit regularization.

Transformer-enhanced designs address long-range dependency limitations of CNNs (117). He et al. proposed HCTNet, incorporating attention-based encoding and decoder-side spatial refinement; under cross-validation, they reported consistent improvements on Breast Ultrasound Images Dataset(BUSI) and Dataset B with modest parameter growth (118, 119). To mitigate lesion–background confusion, TaiChiNet adopts complementary “negative–positive” pathways and progressive hard/easy sample learning, improving sensitivity but with substantially increased model capacity (120). Beyond architectural changes, physics-informed regularization has been explored to improve boundary consistency under speckle noise. Ding et al. introduced a multi-scale refinement decoder with a composite loss incorporating total variation like constraints, reporting improved robustness on BUSI/BUSIS (121–123). Notably, claims of very high overlap metrics should be interpreted cautiously when external validation is absent or when preprocessing/augmentation and patient-level data splitting are insufficiently specified.

#### 5.2.2 From segmentation to diagnosis and prognosis

Three integration patterns recur (i) multimodal fusion to enrich representation (B-mode + elastography, etc.), (ii) cascaded designs that use segmentation to focus classification and improve interpretability, and (iii) multi-task frameworks jointly optimizing segmentation and outcome prediction. Misra et al. fused B-mode and strain elastography via a multimodal segmentation–classification pipeline and reported strong sensitivity/specificity on their cohort (124). Cascaded approaches (segmentation → classification) can improve ROI quality and interpretability, e.g., by pairing a segmentation module with a lightweight classifier and reporting Grad-CAM overlays (125, 126). For survival endpoints, Wenwen et al. combined ultrasound-derived features with clinico-pathological variables and reported internal and external validation AUCs for multi-year OS/DFS prediction (127). For clinical deployment, such pipelines should additionally report calibration, decision-curve analysis, and robustness to device/domain shift (8, 89).

#### 5.2.3 Methodological gaps

The dominant barriers remain (i) class imbalance and minority subgroup scarcity, (ii) domain shift across devices, operators, and institutions, and (iii) limited multi-center prospective validation aligned with clinical endpoints. Addressing these requires standardized patient-level splits, explicit external testing, harmonized reporting, and uncertainty-aware decision support rather than accuracy alone.

### 5.3 X-ray mammography and contrast-enhanced mammography

In mammography, AI studies span screening-like detection and diagnostic classification; in practice, the boundary between these tasks is often blurred (5). For TNBC, a central objective is *non-invasive subtype inference* from imaging phenotypes, i.e., a “radiological virtual biopsy” paradigm that seeks to map macroscopic appearances to underlying biology (132). Representative mammography and CEM/CESM studies for TNBC subtyping, treatment response, and prognosis are summarized in **Table 3**. This is intrinsically challenging because TNBC can exhibit less conspicuous mammographic signatures (e.g., fewer calcifications, more benign-appearing margins), which can increase false negatives for both radiologists and models (132).

**Table 3:** Representative mammography-based AI studies for TNBC subtyping, treatment response, and prognosis.

Study	N (Dataset)	Input	Task/Endpoint	Model	Split & CV	Patient-level split	External validation	Endpoint definition	Key results
Luo (2025) (133)	5,966	MG (DM)	Subtyping/subtype	EfficientNet-B5	4176 895 895 (T/V/Ts)	Yes	Yes^a^	IHC (ER/PR/HER2/Ki67)	Cls Acc=0.85; AUC = 0.96
Zhang (2023) (134)	2,906	MG+US+Path	Subtyping/subtype	Multi-modal	2306 600 (T/Ts); NR	Yes	No	IHC status	Cls AUC = 0.95
Niu (2022) (135)	836	MG (DM) + MRI	Subtyping/subtype	Radiomics + SVM	585 251 (T/Ts); NR	Yes	No	IHC confirmation	Cls AUC = 0.86
Wang (2020) (136)	498	MG (DM)	Subtyping/subtype	Radiomics + SVM	348 150 (T/Ts); NR	Yes	No	Pathological IHC	Cls AUC = 0.81
Zhang (2023) (137)	129	MG (CESM)	Response/pCR	Radiomics + LR	90 39 (T/Ts); NR	Yes	No	pCR (Miller-Payne)	Cls AUC = 0.92 AUC=0.91 (Ts)
Ma (2025) (87)	878	MG (CEM)	Subtyping/subtype	Radiomics + SVM	614 264 (T/Ts); NR	Yes	No	IHC status	Cls AUC = 0.93 AUC=0.91 (Ts)
Khalid (2025) (138)	304	MG (DM)	Relapse/relapse	Radiomics + DL	7 3 (T/Ts); NR	Yes	No	Follow-up recurrence	Cls AUC = 0.95

MG, mammography; DM, digital mammography; CESM, contrast-enhanced spectral mammography; CEM, contrast-enhanced mammography; Path, pathology; SVM, support vector machine; V, validation set. [a] Based on multi-center data integration providing high generalization.

Cross-vendor and cross-site generalization is less frequently evaluated than within-cohort performance, limiting immediate clinical transferability.

Deep models (CNNs and attention-enhanced variants) have been applied to ROI-based subtype classification. Luo et al. combined DenseNet121 with Convolutional Block Attention Module (CBAM) and reported interpretability observations via Grad-CAM suggesting that peri-tumoral regions may contribute to TNBC discrimination (133). At the same time, the reported AUCs for TNBC-vs-non-TNBC discrimination remain moderate in many single-view, morphology-only settings, underscoring that mammography alone may be insufficient for robust molecular inference without additional information or stronger priors (133).

Radiomics provides high-throughput quantitative descriptors; deep models can serve as automated feature extractors that complement or replace hand-crafted features (4, 99). Contrast-enhanced mammography (CEM/CESM) further introduces functional information and may improve subtype-related discrimination compared to standard digital mammography in some settings (87). Multi-view fusion (e.g., Cranio-Caudal (CC) + Mediolateral Oblique (MLO)) is another established strategy that better approximates radiologist practice and can reduce single-view ambiguity (84). For segmentation/detection, U-Net variants remain standard; promptable foundation models are increasingly explored, but medical adaptation and rigorous evaluation remain essential (5, 86). Representative digital pathology studies, including WSI-level pCR prediction and nuclei/tissue segmentation tasks, are summarized in **Table 4**.

**Table 4:** Representative digital pathology AI studies relevant to TNBC.

Study	N (Dataset)	Split (T/V/Ts)	Modality	Task	Model	Pt-level	Ext. Valid.	Endpoint definition	Key results
Duanmu (2022) (148)	269	177 92 (Ts)	H&E WSI	Response (pCR)	MIL	Yes	No	pCR No residual invasive cancer	AUC 0.78
Fisher (62) (2024)	164	85 79 (T/V)	H&E WSI	Classification	U-Net	Yes	Yes^a^	Expert consensus on TILs	AUC 0.82
Zeng (149) (2024)	440	261 107 72	Biopsy path.	Classification	DPM	Yes	No	Histological/IHC subtyping	AUC 0.79
Khan (95) (2025)	204	174 30 (Ts)	Histopath.	Classification	MIL	Yes	Yes	Pathologist-verified labels	AUC 0.86
Imtiaz (145) (2023)	764^b^	30 14 (Ts)	H&E Patches	Nuclei Seg.	BAWGNet	Yes	No	Pixel-level manual masks	Dice 0.91
Roy (144) (2024)	94^c^	7 2 1	H&E Patches	Nuclei Seg.	AWGUNet	Yes	No	Manual expert annotation	Dice 0.82
Rong (142) (2023)	1,100	70 15 15	Histopath.	Seg + Cls	HD-YOLO	Yes	Yes	Mask Nuclei; Cls Cell types	mIoU 0.84

DPM, deep pathology model; mIoU, mean intersection over union. [a] Galway cohort validation. [b] Combined DSB, MoNuSeg, and TNBC datasets. [c] MoNuSeg and TNBC nuclei datasets.

Whole-slide pipelines are sensitive to stain/scanner variation; domain normalization and multi-center validation are frequent determinants of real-world performance.

### 5.4 Computational pathology

Histopathology remains the diagnostic gold standard and provides direct access to tumor cellularity, stromal composition, and immune infiltration patterns that are central to TNBC biology. AI for TNBC pathology has advanced rapidly, largely driven by whole-slide imaging (WSI) availability and weak supervision strategies that better match clinical annotation realities (62, 139). We organize this literature around (i) WSI-level prediction under weak supervision (notably NAC response), (ii) segmentation and quantification of histological primitives, and (iii) data constraints and privacy-preserving collaboration.

#### 5.4.1 Weakly supervised prediction of NAC response

Predicting pCR after NAC is clinically impactful and strongly associated with long-term outcomes in TNBC (62). Multiple studies adopt multiple instance learning (MIL) or attention-based MIL to train from slide-level labels, mitigating the prohibitive cost of dense pixel annotations (140, 141). Khan et al. reported an attention-based MIL framework for pCR prediction from pre-treatment H&E WSIs with AUC 0.86 internally and 0.78 externally, and showed that attention maps aligned with immune-related biomarkers (95). Ogier Du Terrail et al. demonstrated federated training of a pCR predictor across institutions, achieving AUC 0.75 under distributional heterogeneity while preserving privacy (140). Despite progress, reproducibility depends on controlling stain/scanner variability, patient-level splitting, and transparent reporting of tile sampling and aggregation choices.

#### 5.4.2 Segmentation and quantitative pathology

Many high-level predictors depend on +robust quantification of nuclei, tumor regions, and tumor-infiltrating lymphocytes (TILs), making segmentation a critical enabling step (142, 143). Recent designs extend U-Net with wavelet-domain features, attention, and boundary-aware modules to improve nuclear delineation under dense cell packing (144, 145). For tissue-level analysis, pipelines that segment tumor– stroma regions and then quantify TILs can support reproducible immune scoring with prognostic relevance (146). Future work should more systematically report how segmentation uncertainty propagates into downstream biomarkers and clinical endpoints.

#### 5.4.3 Data scarcity, privacy, and robustness

Limited curated TNBC pathology cohorts and privacy constraints remain key bottlenecks. Synthetic data generation (e.g., GAN-based augmentation) can expand training diversity, but requires careful validation to avoid distributional artifacts and over-optimistic estimates (147). FL provides a practical route to multi-institutional training without data sharing and has shown improved generalization compared with single-center training (140). A pressing methodological frontier is to combine privacy-preserving training with domain generalization (stain/scanner shifts) and calibrated, uncertainty-aware decision support.

### 5.5 Genomics and transcriptomics modeling

Genomic profiling is fundamental for characterizing TNBC heterogeneity and defining clinically relevant subtypes (e.g., Breast Lesion Image Analysis (BLIA), Breast Lesion Image Segmentation (BLIS), Multiparametric Evaluation Segmentation dataset (MES), LAR) (1, 3). Representative genomics, transcriptomics, single-cell, and multi-omics ML studies for TNBC subtyping and prognosis are summarized in **Table 5**. Early machine learning methods (e.g., regularized regression, SVM/RF) established strong baselines for subtype discrimination and signature discovery (59). Recent work increasingly emphasizes representation learning and multi-omics integration to capture complementary signals and improve generalization (150).

**Table 5:** Representative machine-learning studies using multi-omics data for TNBC subtyping and prognosis.

Study	N (Dataset)	Split (T/V/Ts)	Modality	Task	Model	Pt-level split	Ext. valid.	Endpoint definition	Key results
Kim (2025) (156)	102 (INT)	7 3 (Ts)	Genomics	Relapse	Cox-LASSO	Yes	No	Relapse Recurrence post-chemo (follow-up to 2022)	C-idx 0.69
Yang (2024) (58)	468 (TCGA)	8 2 (Ts)	Multi-omics	Subtyping	DL	Yes	Yes^a^	Subtyping TNBC/non-TNBC (ER/PR/HER2 status)	Acc 0.97
Ren (2024) (154)	606 (TCGA)	8 1 1 (T/V/Ts)	Multi-omics	Subtyping	MV-GNN	Yes	No	Endpoint 1 G1/G2/G3; Endpoint 2 LumA/B/H2+/TNBC	Acc 0.96
Wu (2021) (157)	1105 (TCGA)	8 2 (Ts)	Genomics	Subtyping	RF, SVM	Yes	Yes^b^	Endpoint 1 Subtype/PAM50; Endpoint 2 OS (Dx to death)	Acc 0.98
Bareche (2021) (158)	532 (GEO)	198 334 (Ts)	Microarray	Subtyping	SVM, KNN	Yes	No^c^	Subtyping BLIA/BLIS/MES/LAR (gene-based)	AUC 0.99
Xu (2019) (159)	1570 (SYSMH)	8 2 (Ts)	Genomics+Clin	Mortality	RF, GBM	Yes	No	Death Discharge to death (5-year follow-up)	AUC 0.90
Liu (2023) (152)	680 (MIX)	Cohort split	scRNA-seq	Prog/Sub	CM1 score	Yes	Yes^d^	Subtyping TNBCtype-4; Prognosis OS/DFS/RFS/MFS	AUC 0.79
Bareche 2 (2020) (3)	151 (MIX)	Cohort split	Transcriptomic	Prognosis	Log/Cox Reg	Yes	Yes^e^	OS/RFS Diagnosis to death/relapse	HR reported

INT, internal; LASSO, least absolute shrinkage and selection operator; MV-GNN, multi-view graph neural network; RF, random forest; GEO, Gene Expression Omnibus; KNN, k-nearest neighbors; GBM, gradient boosting machine; MIX, mixed cohorts; scRNA-seq, single-cell RNA sequencing; RFS, relapse-free survival; MFS, metastasis-free survival; C-idx, concordance index; SYSMH, Sun Yat-sen Memorial Hospital; CM1 score, TNBC subtype and prognosis assessment score based on transcriptomic profiles.

High-dimensional molecular modeling requires rigorous batch correction, stability analysis, and independent validation to produce reproducible signatures.

Methodologically, graph neural networks (GNNs) operationalize systems-biology intuition by modeling patient similarity networks rather than treating samples as independent points (151). Transformer-based attention has also been explored to capture non-local gene module dependencies in high-dimensional expression profiles (94). Alongside performance, explainable AI has become standard to identify genes and pathways driving predictions, although stability of explanations across cohorts and preprocessing pipelines remains an important concern (97).

Emerging molecular data streams can address several limitations of bulk transcriptomics. Single-cell RNA sequencing can separate malignant, immune, and stromal cell states and has been used to derive TNBC subtype-specific prognostic signatures (152). Spatial transcriptomics adds tissue context by preserving the geographic organization of tumor, stromal, and immune compartments; recent TNBC work has shown substantial spatial heterogeneity with potential clinical implications (153). Graph-based multi-omics integration can encode patient, gene, pathway, or cell-state relationships across omics layers, offering a principled way to model tumor programs and microenvironmental interactions (151, 154).More recently, molecular foundation models such as scGPT have begun to learn transferable representations from large-scale single-cell and multi-omics corpora (155).These approaches are promising for biomarker discovery and mechanism-informed stratification, but they remain limited by batch effects, sparse measurements, small TNBC-specific cohorts, and the lack of prospective clinical validation.

Importantly, genomics-only models capture molecular states but do not directly encode spatial phenotype or microenvironmental architecture. Consequently, a novel approach is to treat genomic modeling as one component of a broader patient representation that benefits from cross-modal integration (see Section Multimodal learning and fusion strategies).

## 6 Multimodal learning and fusion strategies

### 6.1 Why multimodal modeling is clinically motivated

TNBC exhibits pronounced heterogeneity across macroscopic imaging phenotypes, microscopic histology, and molecular programs, such that any single modality captures only a partial view of disease biology. Multimodal learning aims to integrate complementary signals to support clinically actionable endpoints, including molecular subtyping, response prediction under neoadjuvant therapy, and survival or recurrence risk stratification, within realistic decision windows (79, 160). Importantly, reported gains from multimodal fusion should be interpreted through study design and validation level performance improvements may reflect information complementarity, but can also arise from cohort effects, endpoint definitions, and leakage-prone splitting strategies if not explicitly controlled.

### 6.2 Fusion levels and representative design patterns

Methodologically, multimodal fusion is commonly categorized into early, intermediate, and late fusion, which differ in their assumptions about alignment and data availability (79, 84).

- Early fusion concatenates modality-specific inputs (or low-level features) and learns a joint representation end-to-end. This strategy is appealing when modalities are well-aligned (e.g., paired imaging sequences in MRI) and missingness is limited, but may be sensitive to acquisition variability and modality imbalance.
- Intermediate fusion learns modality-specific embeddings first and then performs cross-modal interaction via attention, gating, or cross-modal alignment modules. This is currently the most prevalent paradigm because it preserves modality-specific inductive biases (e.g., 3D CNNs for volumetric MRI; MIL/Transformer encoders for WSI) while enabling controlled interaction.
- Late fusion combines outputs from separate unimodal models (e.g., weighted ensembling or stacking). It is often easier to train under heterogeneous data availability and can be robust to missing modalities, but may underutilize fine-grained cross-modal correspondences.

Across reported TNBC studies, intermediate and late fusion are frequently preferred in practice due to heterogeneous data completeness and the need to incorporate structured clinical variables alongside imaging or pathology.

### 6.3 Clinical problems addressed by multimodal fusion

Rather than cataloging studies by modality alone, it is more informative to organize multimodal evidence around *clinical questions* while making *fusion strategy* and *validation strength* explicit.

#### 6.3.1 Molecular subtyping and diagnostic enrichment

Most work in this category uses *intermediate* or *late* fusion of paired radiology modalities (commonly mammography + ultrasound) to predict molecular subtype or enrich diagnostic assessment. Reported gains are typically based on retrospective internal splits; external validation across devices or institutions is less consistently documented. As an example, Multi-modal Deep Learning with Intra- and Inter-modal Attention (MDL-IIA) integrates mammography and ultrasound via intra-/inter-modal attention and reports improved subtype prediction compared with radiological assessment for certain subtype distinctions (134). For translation, the key evidentiary requirement is cross-site evaluation under scanner/protocol shift with strict patient-level splitting.

#### 6.3.2 Treatment response prediction under NAT/NAC

Response prediction (especially pCR) is the most directly actionable multimodal application and usually follows one of two patterns.

- Longitudinal radiology fusion combines baseline and on-treatment MRI to encode therapy-induced dynamics (conceptually aligned with delta-radiomics). Multi-stage MRI fusion has shown strong performance with external evaluation in some settings (103).
- Cross-scale fusion integrates radiology with pathology and clinicopathological variables to jointly represent macroscopic phenotype and microscopic ecology. Systems such as Multi-modal Information.

Fusion and Assisted Prognosis System (MIFAPS) combine pretreatment MRI, WSI, and clinical risk factors, suggesting that pathology can add complementary microenvironmental information beyond imaging alone (96).

In addition, radiomics–clinical models are often distilled into nomograms to improve usability, with multimodal ultrasound/radiomics/clinical combinations repeatedly reporting improvements over single modality baselines (57, 127, 161).

#### 6.3.3 Survival and recurrence risk stratification

For DFS/OS/recurrence, multimodal pipelines commonly fuse imaging-derived deep features or radiomics with clinical covariates, and in some studies relate model saliency regions to RNA-seq or pathway signals for interpretability. A representative example is an MRI-based multimodal recurrence framework that links model-derived regions to RNA-seq correlates (80). Such associations are typically observational; their interpretive value depends on cohort representativeness and harmonized preprocessing, and should not be over-interpreted as causal evidence.

### 6.4 Evidence grading validation level and reporting quality

Multimodal gains should be interpreted with explicit attention to validation strength. In this review, evidence can be read through a pragmatic three-tier lens.

- Tier 1 (highest) independent external validation (institutional, temporal, or device/stain shift), preferably multi-center, with patient-level splits and transparent endpoint definitions.
- Tier 2 internal validation with rigorous patient-level splitting and leakage control, plus sensitivity analyses (e.g., missing-modality robustness, subgroup performance).
- Tier 3 single-center retrospective studies with limited reporting of splitting, endpoints, calibration, or shift robustness.

Most TNBC multimodal studies fall in Tier 2–3, with fewer Tier 1 evaluations. FL in pathology provides a realistic pathway to build multi-institutional evidence under privacy constraints (140). Systematic reviews report average improvements of multimodal over unimodal baselines, but heterogeneous endpoint definitions, inconsistent calibration reporting, and incomplete shift analyses continue to limit cross-study comparability (63, 79).

### 6.5 Methodological risks and practical recommendations

From a deployment perspective, the main failure modes are frequently methodological rather than architectural. Key risks include (i) **patient-level leakage** in patch/tile-based pathology and multi-view imaging; (ii) **endpoint ambiguity** (e.g., varying pCR criteria and follow-up definitions); (iii) **missing modality bias**, where models are trained on complete cases but deployed on partially observed patients; and (iv) **domain shift** across scanners, staining protocols, and acquisition parameters. For multimodal TNBC studies intended for clinical translation, reporting should explicitly specify (a) patient-level splitting, (b) endpoint operational definitions, (c) at least one independent external validation or temporally separated cohort, (d) calibration and decision-oriented evaluation, and (e) robustness under modality missingness and protocol shifts. These criteria materially improve the interpretability and trustworthiness of multimodal evidence and clarify the pathway from retrospective accuracy to clinical utility.

## 7 Clinically actionable prediction treatment response and survival

TNBC lacks established endocrine targets and, for many patients, remains primarily managed with cytotoxic chemotherapy, particularly in the neoadjuvant setting (162, 163). Compared with other breast cancer subtypes, TNBC is associated with higher recurrence risk and a greater propensity for visceral and brain metastases, which collectively contribute to poorer overall survival (OS) (1, 112). These clinical realities motivate *time-sensitive* prediction tools that can inform decisions at actionable junctures (e.g., pre-treatment, early during neoadjuvant therapy, and perioperative risk reassessment), rather than solely *post hoc* prognostication.

The central barrier to reliable prognostic assessment is heterogeneity across clinical presentation, pathology, and molecular programs patients with similar stage and routine biomarkers can experience markedly different outcomes (3, 5). Anatomy-driven systems such as AJCC TNM staging offer a necessary baseline but do not explicitly encode molecular biology or microenvironmental state, limiting individualized risk stratification in TNBC (2). Consequently, a major unmet need is the development of models that can (i) robustly identify patients likely to benefit from treatment intensification versus de-escalation, and (ii) provide mechanistically interpretable signals that support clinical reasoning and hypothesis generation (164, 165).

With rapidly expanding clinical, imaging, and omics data streams, machine learning (ML) and deep learning (DL) methods have been explored to extract prognostic information from high-dimensional and heterogeneous inputs, often capturing non-linear interactions that may be missed by conventional regression pipelines (4). This section reviews clinically actionable prediction in TNBC across four complementary axes (1) genomics-based signatures and mechanism-informed regulatory models; (2) radiomics and longitudinal (delta) imaging phenotypes for response and survival; (3) computational pathology for decoding tumor microenvironment (TME) structure and treatment sensitivity; and (4) multimodal fusion frameworks that integrate multi-scale data into unified predictive systems. A cross-modality summary of ML/DL prognostic studies, grouped by primary data modality and endpoint, is provided in **Table 6**. Throughout, emphasis is placed on (a) endpoint clarity (pCR/DFS/OS definitions), (b) evaluation rigor (patient-level splits, external validation), and (c) translational constraints (standardization, domain shift, and interpretability).

**Table 6:** Summary of machine-learning/deep-learning studies for TNBC prognosis, grouped by primary data modality.

Study	N (Dataset)	Split (T/V/Ts)	Modality	Task	Model	Pt-level split	Ext. valid	Endpoint definition	Key results
Xu (2025) (65)	37,982 (SEER)	65 17.5 17.5	Clinical	Prognosis	N-MTLR	Yes	Yes^a^	OS Dx to death; Mean follow-up 34m	AUC 0.82
Kris (2023) (183)	243 (INT)	165 78 (T/Ts)	Pathology	pCR	CNN	Yes	No	pCR ypT0/Tis+ypN0	AUC 0.75
Kim (2025) (156)	102 (INT)	7 3 (T/Ts)	Genomics	Relapse	RF + LR	Yes	No	Relapse Post-chemo recurrence	AUC 0.91
Huang (2023) (181)	84 (INT)	64 20 (T/Ts)	Pathology	pCR	LR	Yes	Yes^b^	pCR No residual invasive cancer + node-	AUC 0.77
Terrail (2023) (140)	686 (MULTI)	449 237 (T/Ts)	Pathology	pCR	Fed-WSI	Yes	Yes^c^	pCR RCB 0; DFS/OS Relapse/death	AUC 0.66
Fisher (2024) (62)	164 (INT)	85 79 (T/Ts)	Pathology	pCR	RBF-SVM	Yes	Yes^d^	pCR No residual invasive disease	AUC 0.83
Bai (2021) (180)	920 (MIX)	171 749 (T/Ts)	Pathology	Prognosis	TIL-NN	Yes	Yes^e^	OS Dx to death; Med. follow-up 64m	HR 0.49

Dx, diagnosis; Med, median; RCB, residual cancer burden; Fed-WSI, federated whole-slide imaging; N-MTLR, regularized multi-task logistic regression; TIL-NN, tumor-infiltrating lymphocyte based neural network; m, months; MULTI, multi-center.

Most evidence remains single-modality with heterogeneous endpoints; multimodal and externally validated prognostic models are still comparatively scarce.

### 7.1 Genomic features

Genomic and transcriptomic profiles provide a principled basis for modeling TNBC heterogeneity and prognosis (162). Classical transcriptome-derived subtype taxonomies (e.g., Lehmann-style schemes) reveal meaningful biological diversity, yet the separation in recurrence-free survival (RFS) between subtypes can be modest, limiting direct clinical stratification utility in some settings (5, 166).

Methodologically, genomics studies face persistent challenges the *small-N, large-P* regime, platform/batch effects, and confounding variation due to tumor purity and cellular composition. These factors can destabilize feature selection and impair cross-cohort reproducibility if not explicitly addressed (e.g., batch correction and deconvolution) (167–169).

ML pipelines are frequently used to identify parsimonious prognostic signatures from gene expression or NGS data by combining feature screening with regularized modeling and internal validation. Kim et al. reported a two-stage workflow (RF screening followed by LR backward selection) and proposed a five-gene signature comprising fibroblast growth factor 10 (*FGF10*), GATA binding protein 1 (*GATA1*), dehydrogenase E1 and transketolase domain containing 1 (*DHTKD1*), T-box transcription factor 21 (*TBX21*), and the ret proto-oncogene (*RET*), achieving an AUROC of 0.9087 in an internal test set (156).

While such studies illustrate feasibility, their translational strength depends critically on external validation across independent cohorts and sequencing platforms, as well as transparent reporting of batch correction, feature stability, and leakage-avoidance practices.

Regularization, ensemble learning, and resampling-based stability assessment are commonly employed to mitigate overfitting, but their effectiveness remains contingent on cohort size and signal-to-noise characteristics (9, 170).

Beyond sparse signatures, DL models can learn low-dimensional latent representations that summarize multi-omics data while preserving outcome-relevant signals.

Autoencoder-based frameworks are widely used to compress heterogeneous molecular modalities (e.g., mRNA, miRNA, methylation, CNV) and feed learned embeddings into survival models or boosting-based predictors. The Dense Contextual Attention Pooling (DCAP) framework employs a denoising autoencoder to learn latent features from multi-omics inputs, followed by gradient boosting for survival prediction and downstream identification of prognostic gene sets (150).

In practice, interpretability often relies on *post hoc* attribution (e.g., SHAP (SHapley Additive exPlanations)) and pathway enrichment, and performance is sensitive to cohort size, hyperparameters, and missing-modality patterns (171).

A comparatively underexplored approach converts transcriptomic vectors into artificial image objects (AIOs) and applies CNNs to capture co-expression patterns under spatial inductive biases. Chen et al. adopted this strategy (CECE), using saliency maps to derive a 21-gene candidate set and evaluating risk stratification across multiple datasets (166, 172).

Because the spatial layout is defined by encoding rules rather than biology, robustness checks (e.g., varying arrangements and permutation tests) are essential to demonstrate that findings are not artifacts of representation design.

To move beyond purely correlational signatures, mechanism-driven frameworks incorporate known pathway structure and regulatory logic. Garrone, La Porta, and colleagues proposed ARIADNE, a Boolean network model of EMT regulation (72 genes) that simulates dynamical behavior to generate a continuous phenotypic landscape spanning epithelial, mesenchymal, and hybrid E/M states (5, 173). Mapping patient transcriptomes onto this landscape yields quantitative scores intended to reflect invasive potential and treatment responsiveness.

Such models are attractive for interpretability and hypothesis testing, but require careful validation across cohorts and, ideally, experimental corroboration of predicted state transitions and pathway perturbation effects.

### 7.2 Radiomic features

Radiomics offers a non-invasive framework for quantifying spatial tumor heterogeneity from medical imaging (e.g., MRI, ultrasound, CT, PET (positron emission tomography)), extracting high-throughput descriptors of morphology, texture, and signal statistics within intra- and peri-tumoral regions (99, 174). With ML/DL advances, radiomics has expanded from single time-point characterization to longitudinal analysis (delta-radiomics), capturing treatment-induced changes in imaging phenotype that may anticipate response and long-term outcome (60, 103).

#### 7.2.1 Static radiomics for heterogeneity-linked risk stratification

Imaging phenotypes can reflect underlying molecular programs and TME composition. For example, Jiang et al. reported a DCE-MRI peritumoral study-defined radiomic feature, Peri_V_DN (peritumoral variance of dependence non-uniformity; a GLDM (Gray Level Dependence Matrix)-derived descriptor defined as the variance among MRI sequences of dependence non-uniformity extracted from peritumoral ROIs), which was associated with RFS/OS and linked to immunosuppression-related pathways and metabolic gene programs via radiogenomic analysis (175). Such associations are promising but remain sensitive to acquisition protocols, reconstruction, preprocessing (registration/normalization), and segmentation variability. Therefore, external validation across centers/vendors and explicit standardization strategies are prerequisites for credible generalization claims. Reviews have summarized radiomics-based nomograms with external validation performance in clinically relevant ranges (C-index ≈ 0.71–0.73; AUC ≈ 0.81–0.85), yet many studies remain retrospective and single-center, limiting translational readiness (4, 132).

#### 7.2.2 Delta-radiomics and longitudinal imaging for pCR and survival

Pathologic complete response (pCR) after neoadjuvant therapy (NAT) is a widely used surrogate endpoint linked to favorable prognosis in TNBC (85, 132). Longitudinal radiomics aims to provide early, non-invasive response assessment before surgery. In a multi-center study, Huang et al. integrated multi-parametric MRI features from pre- and post-NAT stages and reported that stacking pre-, post-, and delta-features achieved strong pCR prediction, including robust performance in external cohorts (103). These results support the broader principle that treatment-induced changes in perfusion/texture/morphology can encode response dynamics beyond baseline phenotype alone; however, clinical deployment requires standardized time points, high follow-up compliance, and reliable registration/segmentation to avoid noise amplification.

Some studies focus on baseline-only prediction to reduce operational burden. Xu et al. developed a 3D DL model using baseline multi-parametric MRI (DCE, DWI) plus clinicopathological variables, evaluating architectural choices, ROI definitions, and tumor volume preprocessing; fusion of imaging and clinical variables improved predictive performance, while tumor volume preprocessing exerted a comparatively larger effect than specific backbone variants (65). Such findings suggest that careful control of input definition and preprocessing may yield greater gains than marginal architectural changes, with implications for pragmatic clinical workflows.

#### 7.2.3 Metabolic imaging (FDG-PET/CT) as a complementary axis

FDG-PET/CT provides metabolic descriptors (e.g., SUV, MTV, TLG) that can complement structural/functional MRI features in multimodal predictors. Reported models combining PET/CT metabolic features with other biomarkers have achieved moderate-to-strong external pCR prediction performance (e.g., AUC around 0.82 in some settings) (176). Translation is constrained by cost and accessibility, and by remaining variability in semi-quantitative parameters across centers despite protocol recommendations; harmonization and vendor-robust thresholds remain active challenges (177, 178).

### 7.3 Decoding the tumor microenvironment

TNBC outcomes are strongly shaped by the TME, whose cellular composition and spatial organization influence progression, metastasis, and treatment sensitivity (62). Conventional assessment (e.g., manual TIL scoring) is informative but subject to inter-observer variability and limited granularity (60, 179). Computational pathology extends TME assessment from semi-quantitative counts to scalable, high-dimensional modeling of cell states, spatial ecology, and microenvironmental “ecotypes”.

#### 7.3.1 Automated cell quantification and spatial interaction modeling

Automated TIL quantification and cell typing have been linked to invasive disease-free survival (iDFS) and immunotherapy response in multiple settings, though reproducibility depends on annotation standards and algorithmic calibration (179, 180). Graph-based spatial modeling treats cells or regions as nodes and encodes proximity or interaction patterns; reported associations include improved pCR likelihood when tumor cells are spatially proximal to TILs, and adverse outcomes under specific vascular–tumor configurations (62). For translation, the interpretability of complex spatial metrics and the representativeness of biopsy samples (sampling bias) must be explicitly considered.

#### 7.3.2 Predicting NAT response from pretreatment pathology and federated learning

Pretreatment biopsy WSIs can encode response-relevant morphology and immune context. Ogier du Terrail et al. conducted a multi-center FL study predicting NAT response from H&E images while preserving data privacy, and highlighted interpretable cues (e.g., hemorrhage, necrosis, TILs) that generalized across cohorts (140). Huang et al. integrated H&E with multiplex immunohistochemistry and reported immune-state markers (e.g., Programmed Death-Ligand 1 (PD-L1) within lymphocyte aggregates) associated with pCR (181). Methods that avoid explicit cell segmentation, such as SparTile, model tile-level protein co-expression patterns to define microenvironment ecotypes and correlate them with survival across cohorts (182). Together, these approaches provide a quantitative basis for linking spatial TME organization to therapy response and prognosis, while underscoring the need for stain/scanner robustness, cohort diversity, and explicit external validation.

### 7.4 Clinical translation pathways

Clinical translation requires more than discrimination models must be calibrated, robust to domain shift, operationally feasible, and interpretable at decision points. A practical pathway is (i) risk stratification from routinely available data, (ii) regimen-specific response prediction, and (iii) mechanism-aware refinement that supports intervention hypotheses.

#### 7.4.1 From staging to data-driven risk stratification

Large-scale tabular cohorts enable refined survival prediction beyond AJCC staging. Using SEER data, Xu et al. trained an N-MTLR-based survival model and reported improved C-index versus Cox and random survival forests, and derived a risk staging system with better AUC than AJCC TNM (65). For clinical adoption, such models should additionally report calibration, time-dependent performance, and decision-focused evaluation (e.g., decision curve analysis), and be validated across geographic and practice-setting shifts.

#### 7.4.2 Pretreatment response prediction to support treatment matching

Because pCR is prognostically meaningful in TNBC, pretreatment predictors can directly inform therapy selection. Krishnamurthy et al. used CNN-derived WSI features combined with clinical variables to predict pCR, reporting AUC improvements in specific subgroups (183). Sturm et al. reported similar trends compared to conventional clinicopathological predictors (184). Translational readiness hinges on robustness to staining/scanning variation, clarity of endpoint definition, and workflow integration (turnaround time, failure handling, and quality control).

#### 7.4.3 Mechanism-informed models for interpretability and hypothesis testing

Mechanism-driven frameworks such as ARIADNE aim to connect transcriptomic state to EMT-driven plasticity and treatment resistance, offering interpretable scores linked to aggressiveness and clinical outcomes (173). Before deployment, such models require iterative validation on independent cohorts and experimental corroboration to ensure that mechanistic assumptions remain consistent with real-world tumor biology.

### 7.5 Multimodal fusion frameworks

Because each modality captures only a slice of TNBC biology, multimodal fusion has become a central strategy to improve robustness and clinical utility (79, 84). Imaging provides spatial phenotype, pathology captures micro-scale tissue organization, clinical variables encode patient context, and liquid biopsy/omics can reflect temporally evolving disease state. Multimodal systems typically adopt modality-specific encoders (e.g., 3D CNNs for MRI, CNN/ViT for WSI, embeddings for tabular data) and integrate representations via attention, gating, or decision-level ensembles (79).

Representative results include Ben Rabah et al. reporting substantial gains when augmenting mammography-based prediction with clinical metadata (185); Yu et al. developing an MRI-based multimodal recurrence stratification framework with external evaluation and biological correlation via RNA-seq (80); and Lyu et al. integrating ultrasound-based multimodal features with external testing (57). In pathologycentric prognostic modeling, integrating deep WSI features with clinical covariates within survival networks can outperform unimodal baselines (151). Liquid biopsy (e.g., ctDNA) provides complementary temporal information for MRD and early recurrence risk, and joint modeling with imaging phenotypes is increasingly explored, though results depend on sampling schedules, assay sensitivity, and cross-platform harmonization (186, 187).

Systematic evidence suggests that multimodal models often outperform unimodal counterparts, primarily due to complementary information across modalities rather than architectural novelty alone (79). Nonetheless, reported gains must be interpreted under evaluation realism patient-level splits, explicit missing-modality handling, external/multi-center validation, calibration, and transparent reporting of failure modes. Prototype platforms (e.g., clinical-facing tools built around recurrence models) are encouraging, but prospective studies remain essential to establish impact on decisions and outcomes (80).

## 8 Discussion

### 8.1 Cross-modal synthesis of current evidence

Across radiology, pathology, and omics, current evidence supports a multi-scale view of TNBC radiology captures macroscopic phenotype and therapy-induced dynamics; pathology quantifies cellular composition and microenvironmental architecture; and omics interrogates molecular programs and regulatory states (1). Multimodal models tend to outperform unimodal baselines when each modality contributes non-redundant signal, but reported gains are heterogeneous and may attenuate under external shift, stricter endpoint definitions, or stronger leakage control (79, 84). Accordingly, performance summaries should be interpreted in the context of cohort design (single- vs multi-center), validation level (internal vs external), and transparency of preprocessing and splitting.

### 8.2 Clinically robust signals and evidence boundaries

The approaches that currently appear most clinically robust are not necessarily the most complex architectures. Longitudinal MRI and delta-radiomics for neoadjuvant response prediction are clinically plausible because they measure treatment-induced change with in an action able window, and some studies report external testing (103). Computational pathology models that quantify TILs, spatial immune organization, or pretreatment WSI features are also promising because they align with known TNBC immunobiology and can be evaluated on routinely available biopsy material (62, 140). Radiomics–clinical orpathology–clinical fusion models maybe more deployable than highly complex multimodal systems when they use routinely collected inputs, provide calibrated risk estimates, and are externally evaluated.

By contrast, several high-performing models should be interpreted as hypothesis-generating rather than clinically established. Some studies use mixed breast cancer cohorts in which TNBC represents a subgroup, so their overall performance cannot be assumed to apply to TNBC without subtype-specific testing. Omics-only signatures, graph-based molecular models, and spatial/single-cell approaches offer strong biological insight, but they remain vulnerable to small sample sizes, batch effects, platform differences, and limited prospective validation. This distinction between TNBC-exclusive, TNBC-enriched, and broader breast-cancer evidence is important when judging whether a model is ready for clinical translation.

### 8.3 What limits translation in practice

Translation is more often limited by evidence generation than by network design. Recurring weaknesses include (i) small or single-center cohorts with limited demographic and scanner/stain diversity; (ii) endpoint ambiguity (inconsistent pCR definitions, follow-up windows, and censoring rules); (iii) leakage risks, especially in patch-based pathology and multi-view imaging when splits are not strictly patient-level; (iv) missing-modality mismatch between training (complete cases) and deployment (partially observed patients); and (v) limited external validation and incomplete reporting of calibration, uncertainty, and failure modes (4, 85). These issues reduce clinical reliability even when retrospective discrimination metrics appear competitive.

### 8.4 Reporting, bench marking, and reproducibility standards

Transparent reporting is a prerequisite for comparing TNBC AI studies. Prediction-model studies should align with TRIPOD+AI, especially for participant flow, predictor definition, outcome definition, sample size, missing data, model development, and external evaluation (188, 189). Imaging AI studies should additionally report items emphasized by CLAIM, including reference standards, data partitions, preprocessing, model specification, performance uncertainty, and whether testing is internal or external (190). If an AI tool is prospectively tested as an intervention, CONSORT-AI becomes relevant for reporting human-AI interaction, input data handling, failure cases, and integration into the clinical pathway (191).

Public resources such as TCGA, GEO, SEER, DUKE/I-SPY2-derived imaging cohorts, and institutional WSI collections can support benchmarking, but they differ in inclusion criteria, treatment era, endpoint definitions, image protocols, molecular platforms, and follow-up completeness. Future benchmark studies should therefore report cohort provenance, patient-level linkage, preprocessing versions, exclusion criteria, and external test-site characteristics. Code availability, model cards, datasheets, inference settings, and documented constraints on data sharing would substantially improve reproducibility and help distinguish true model robustness from dataset-specific optimization (192–194).

### 8.5 Recommendations for high-confidence TNBC AI studies

To strengthen reproducibility and clinical interpretability, future studies should prioritize the following practices.

- Cohort and endpoint transparency state inclusion/exclusion criteria, index time, follow-up window, censoring rules, and missing-data handling; define endpoints (pCR/DFS/OS) in operational terms that can be audited (188, 195).
- Patient-level evaluation enforce patient-level splits for all modalities; for WSI, ensure slide/tile extraction does not introduce cross-split leakage; report the split strategy explicitly (85).
- External validation and shift analysis test on at least one independent cohort (institutional, temporal, or device/stain shift); quantify performance degradation and analyze error patterns by site and subgroup (8).
- Clinical utility beyond discrimination report calibration (and recalibration if needed), decision-oriented evaluation at clinically meaningful operating points, and uncertainty estimates to support safe deployment (97).
- Interpretability with verification accompany attribution maps or prototype retrieval with sanity checks, robustness tests, and where feasible, reader studies or pathology/radiology concordance analysis (196–198).
- Open and reproducible pipelines provide preprocessing details, feature extraction settings, and inference configuration; release code and model cards when permitted, or explicitly state constraints (190, 192–194).

### 8.6 Promising directions

Several directions are likely to shape the next phase of TNBC AI research. Self-supervised and large-scale pretraining can reduce dependence on TNBC-specific labels and improve transfer across institutions and vendors (199, 200). Privacy-preserving multi-center learning, including FL, offers a practical path to scaling training without centralizing sensitive data, but requires careful evaluation under non-IID (non-independent and identically distributed) clinical distributions (140, 201). Domain generalization and harmonization will be essential for imaging and pathology pipelines to remain stable under protocol/stain/scanner shifts (202). Beyond prediction, mechanism-informed modeling (e.g., integrating pathway priors or regulatory-network constraints) can improve interpretability and may support hypothesis-driven stratification (151), while causal and counterfactual frameworks provide a route from risk prediction to decision support under treatment-selection bias (203). Finally, rigorous prospective validation, workflow-aware deployment studies, post-deployment monitoring, and clear accountability for model failure are indispensable to demonstrate clinical benefit, define actionable thresholds, and meet regulatory expectations for AI-assisted oncology tools.

### 8.7 Conclusion

AI has become a central methodology for quantitative TNBC research, enabling multi-scale characterization from imaging, pathology, and omics. The strongest near-term clinical value will come from models anchored to actionable decision points (e.g., early response-adaptive escalation/de-escalation during neoadjuvant therapy and postoperative recurrence-risk stratification) and supported by evidence that prioritizes generalization, calibration, and transparent failure analysis alongside discrimination (132).

Looking forward, multimodal integration is likely to be most beneficial when evaluated under real-world constraints (missing modalities, protocol/stain/scanner shift, and heterogeneous endpoint definitions) using independent external cohorts. Continued progress will depend on multi-institutional evidence, harmonized endpoints, and prospective or pragmatic validation that clarifies how model outputs translate into clinical workflows and patient outcomes.
